# Supplementary material for: Deep learning-based polygenic risk analysis for Alzheimer’s disease prediction
Source: Commun Med (Lond). 2023 Apr 6;3:49. doi: 10.1038/s43856-023-00269-x (PMC10079691; doi:10.1038/s43856-023-00269-x)
Supplement: Supplementary file 1 — Supplementary Information [file 43856_2023_269_MOESM1_ESM.pdf]

Deep learning-based polygenic risk analysis for Alzheimer's disease prediction

Xiaopu Zhou, Yu Chen, Fanny C.F. Ip, Yuanbing Jiang, Han Cao, Ge Lv, Huan Zhong, Jiahang Chen, Tao Ye, Yuewen Chen, Yulin Zhang, Shuangshuang Ma, Ronnie M.N. Lo, Estella P.S. Tong, Alzheimer's Disease Neuroimaging Initiative, Vincent C.T. Mok, Timothy C.Y. Kwok, Qihao Guo, Kin Y. Mok, Maryam Shoai, John Hardy, Lei Chen, Amy K.Y. Fu, Nancy Y. Ip*

SUPPLEMENTARY INFORMATION

Supplementary Methods 5

Supplementary Figure 1. Performance of the different weighted polygenic risk score models for disease classification accuracy in the European-descent cohorts 7

Supplementary Figure 2. Performance of different prediction models for disease classification accuracy in the European-descent cohorts without validation 8

Supplementary Figure 3. Performance of different prediction models for disease classification accuracy in the European-descent cohorts using the five-fold cross-validation method 9

Supplementary Figure 4. Optimization of the neural network model for classifying Alzheimer's disease risk using an independent cohort for cross-validation 10

Supplementary Figure 5. Performance of different polygenic score models in the European-descent Alzheimer's disease cohorts 12

Supplementary Figure 6. Performance of different prediction models for disease classification accuracy in the European-descent cohorts stratified by ethnic group 13

Supplementary Figure 7. Performance of different prediction models for disease classification accuracy in the European-descent population stratified by sex 14

Supplementary Figure 8. Performance of different prediction models for disease classification accuracy in the European-descent population stratified by age group 15

Supplementary Figure 9. Performance of trans-ethnic prediction models for disease classification accuracy 16

Supplementary Figure 10. Genomic correlations among the polygenic risk scores obtained from the trans-ethnic prediction models in Chinese WGS cohort 1 17

Supplementary Figure 11. Performance of polygenic risk models for classifying Alzheimer's disease risk in the Chinese Alzheimer's disease whole-genome sequencing cohorts 18

Supplementary Figure 12. Comparison of the classification accuracy of modified polygenic risk score models using 37 variants in the Chinese population 19

Supplementary Figure 13. Evaluation of different prediction models using 37 variants for disease classification accuracy in European-descent cohorts and Chinese WGS cohort 1 using the five-fold cross-validation method 20

Supplementary Figure 14. Comparison between models using 37 variants and variants selected by *p*-value thresholds for classifying Alzheimer's disease risk in the European-descent cohorts using the five-fold cross-validation method 21

Supplementary Figure 15. Comparison between models using 37 variants and variants selected by *p*-value thresholds for classifying Alzheimer's disease risk in the Chinese population 22

Supplementary Figure 16. Performance of trans-ethnic prediction models using 37 variants for disease classification accuracy in the European-descent cohorts and WGS1 dataset 23

Supplementary Figure 17. Classification of Alzheimer's disease in the Chinese population using neural network models with different variant sets 24

Supplementary Figure 18. Performance of polygenic risk models for classifying Alzheimer's disease risk in Chinese WGS cohort 2 25

Supplementary Figure 19. Associations between polygenic risk score and cognitive performance in patients with mild cognitive impairment 26

Supplementary Figure 20. Associations between polygenic scores and brain region volumes 27

Supplementary Figure 21. Effects of node numbers in the penultimate layer of the neural network model on the biomarker association analysis 28

Supplementary Figure 22. Interpretation of the polygenic risk effects on the modulation of gene expression 30

Supplementary Figure 23. Design and performance of the graph neural network for disease risk classification 32

Supplementary Table 1. Demographic and clinical characteristics of the study cohorts (for Figure 1) 33

Supplementary Table 2. Numbers of variants used for polygenic score analysis (for Figure 1) 35

Supplementary Table 3. Performance of the weighted polygenic risk score models for disease classification accuracy in the European-descent cohorts (for Figure 1) 36

Supplementary Table 4. Performance of the modified weighted polygenic risk score models for disease classification accuracy in the European-descent cohorts (for Supplementary Figure 1) 37

Supplementary Table 5. Evaluation of different prediction models for disease classification accuracy in the European-descent cohorts without validation (for Supplementary Figure 2) 38

Supplementary Table 6. Evaluation of different prediction models for disease classification accuracy in the European-descent cohorts using the five-fold cross-validation method (for Supplementary Figure 3) 39

Supplementary Table 7. Evaluation of different prediction models for disease classification accuracy in independent European-descent cohorts (for Figures 2a–d, Supplementary Figure 4) 40

Supplementary Table 8. Evaluation of different prediction models for disease classification accuracy in independent European-descent cohorts removing potential duplicate samples (for Supplementary Figure 5) 42

Supplementary Table 9. Evaluation of different prediction models for disease classification accuracy in the European-descent cohorts with respect to different ancestral origins (for Supplementary Figure 6) 43

Supplementary Table 10. Evaluation of different prediction models for disease classification accuracy in the European-descent population stratified by sex (for Supplementary Figure 7) 44

Supplementary Table 11. Evaluation of different prediction models for disease classification accuracy in the European-descent population by age group (for Supplementary Figure 8) 45

Supplementary Table 12. Evaluation of trans-ethnic effects on different prediction models for disease classification accuracy in Chinese WGS cohort 1 measured by the area under the receiver operating characteristic curve (for Supplementary Figures 9, 10) ... 46

Supplementary Table 13. Evaluation of the trans-ethnic effects on different prediction models for disease classification accuracy in Chinese WGS cohort 1 measured by the area under the precision-recall curve (for Supplementary Figures 9, 10) 47

Supplementary Table 14. Evaluation of the trans-ethnic effects on different prediction models for disease classification accuracy in Chinese WGS cohort 2 measured by the area under the receiver operating characteristic curve (for Supplementary Figures 9, 10) ... 48

Supplementary Table 15. Evaluation of the trans-ethnic effects on different prediction models for disease classification in Chinese WGS cohort 2 measured by the area under the precision-recall curve (for Supplementary Figures 9, 10) 49

Supplementary Table 16. Sources of the variants for the replication analysis 50

Supplementary Table 17. Performance of the polygenic score models for disease classification accuracy in the Chinese whole-genome sequencing cohorts (for Figure 3a, Supplementary Figure 11) 51

Supplementary Table 18. Evaluation of different prediction models using 37 variants for disease classification accuracy using the five-fold cross-validation method (for Supplementary Figure 13) 52

Supplementary Table 19. Evaluation of the trans-ethnic effects on different prediction models using 37 variants for disease classification (for Supplementary Figure 16) 53

Supplementary Table 20. Stratification of individual disease risk based on polygenic scores and their associations with phenotypes (for Figure 3d, Supplementary Figure 18) 54

Supplementary Table 21. Association between polygenic scores and cognitive performance (for Figures 3e–h, Supplementary Figure 19) 55

Supplementary Table 22. Associations between polygenic scores and the plasma ATN biomarker panel (for Figures 4a–d) 56

Supplementary Table 23. Summary of protein–protein interaction network analysis for plasma proteins associated with polygenic scores (for Figure 4i) 58

Supplementary Table 24. Cell-type enrichment analysis of the plasma proteins in each cluster (for Figure 5c) 59

Supplementary Table 25. Plasma proteins classified in distinct clusters are enriched in specific blood cell types (for Figures 5c, d) 60

Supplementary Table 26. Summary of protein–protein interaction network analysis for plasma proteins enriched in specific blood cell types (for Figures 5d) 61

Supplementary Table 27. Transcript levels of plasma proteins that are abundant in B cells (for Figures 5d, e) 62

Supplementary References 63

Supplementary Methods

Alzheimer's Disease Neuroimaging Initiative cohort

We obtained genotype and phenotype data from the Alzheimer's Disease Neuroimaging Initiative (ADNI) database (https://adni.loni.usc.edu/). The ADNI, led by Michael W. Weiner, MD, was launched in 2003 as a public–private partnership. It is a four-stage study that aims to examine the brain's structure and function, aided by biomarker and clinical data in people aged 55–90 years from the United States and Canada. For the present study, we included array genotype data obtained from ADNI-1, ADNI-2/GO, and ADNI-3 for analysis. After prefiltering, imputation, and postfiltering, we retained 1,382 subjects (n = 689 patients with Alzheimer's disease [AD] and 693 cognitively normal controls [NCs]) for downstream analysis. The phenotypes of the ADNI subjects are from the subjects' latest diagnostic records (updated January 2021).

National Institute on Aging Alzheimer's Disease Centers cohort

The clinical and neuropathology cores of the 29 National Institute on Aging (NIA)-funded Alzheimer's Disease Centers (ADCs) recruited and evaluated autopsy-confirmed and clinically confirmed patients with AD as well as cognitively normal elderly subjects. We retrieved the genotype and phenotype data of this AD cohort (n = 6,065) from the National Institutes of Health (NIH) database of Genotypes and Phenotypes (dbGaP) (accession number: phs000372.v1.p1). Genotype information was generated from the Illumina Human660W-Quad BeadChip or HumanOmniExpress Array. All autopsied subjects were ≥60 years old at death. Dementia in AD was determined according to the Diagnostic and Statistical Manual of Mental Disorders, Fourth Edition (DSM-IV) criteria or a Clinical Dementia Rating ≥1. Further details can be found in publications arising from the corresponding dbGaP project[1,2]. After prefiltering, imputation, and postfiltering, we retained 5,692 subjects (3,946 patients with AD and 1,746 cognitively NCs) for downstream analysis.

Late Onset Alzheimer's Disease Family Study cohort

The Late Onset Alzheimer's Disease (LOAD) Family Study recruited families with two or more siblings with late-onset AD as well as age- and ethnicity-matched, unrelated, nondemented controls. Patients with definite AD were diagnosed according to established neuropathological criteria (i.e., CERAD, Braak, Khachaturian, NIA-RI, or other established criteria). Probable AD or possible AD was ascertained according to the NINCDS-ADRDA (National Institute of Neurological and Communicative Diseases and Stroke/Alzheimer's Disease and Related Disorders Association) criteria. We recruited patients with AD with an age of onset or age at diagnosis ≥50 years old and NCs ≥50 years old. We retrieved genotype and phenotype data from the NIH dbGaP (accession number: phs000168.v2.p2). Individual genotypes were generated from the Human 610Quadv1_B Beadchips (Illumina). After prefiltering, imputation, and postfiltering, we retained 4,278 subjects (n = 2,046 patients with

AD and 2,232 NCs) for downstream analysis. Further details can be found in a publication arising from the corresponding dbGaP project[3].

Filtering and imputation for the array datasets

We converted array genotype information from the ADNI, LOAD, and ADC datasets from PLINK to VCF format using vcfCooker (v1.1.1; https://genome.sph.umich.edu/wiki/VcfCooker). We applied prefiltering at both the individual and variant levels, retaining individuals with a sample call rate ≥95% and variants with a genotype call rate ≥80% separately for each dataset. We submitted the filtered genotype data to the TOPMed Imputation Server[4] (https://imputation.biodatacatalyst.nhlbi.nih.gov) using the TOPMed Imputation Reference panel (TOPMed R2)[5] for phasing and imputation in the form of chromosome-separated VCF files generated by Eagle (v2.4)[6]. We performed post-imputation filtering by removing imputed variants with an imputation $r^2 < 0.4$ using the *bcftools filter* function. We further annotated the dbSNP ID (v154) using the *bcftools annotate* function. We retained single nucleotide polymorphisms with matched dbSNP IDs and alleles for subsequent polygenic score analysis. For part of the quality control analysis, we used PLINK to estimate the identity-by-descent (IBD) using variants with a minor allele frequency (MAF) > 0.01 and pruning according to an R^2 of 0.2, and excluded potentially duplicated subjects according to an IBD > 0.90.

Supplementary Figure 1. Performance of the different weighted polygenic risk score models for disease classification accuracy in the European-descent cohorts

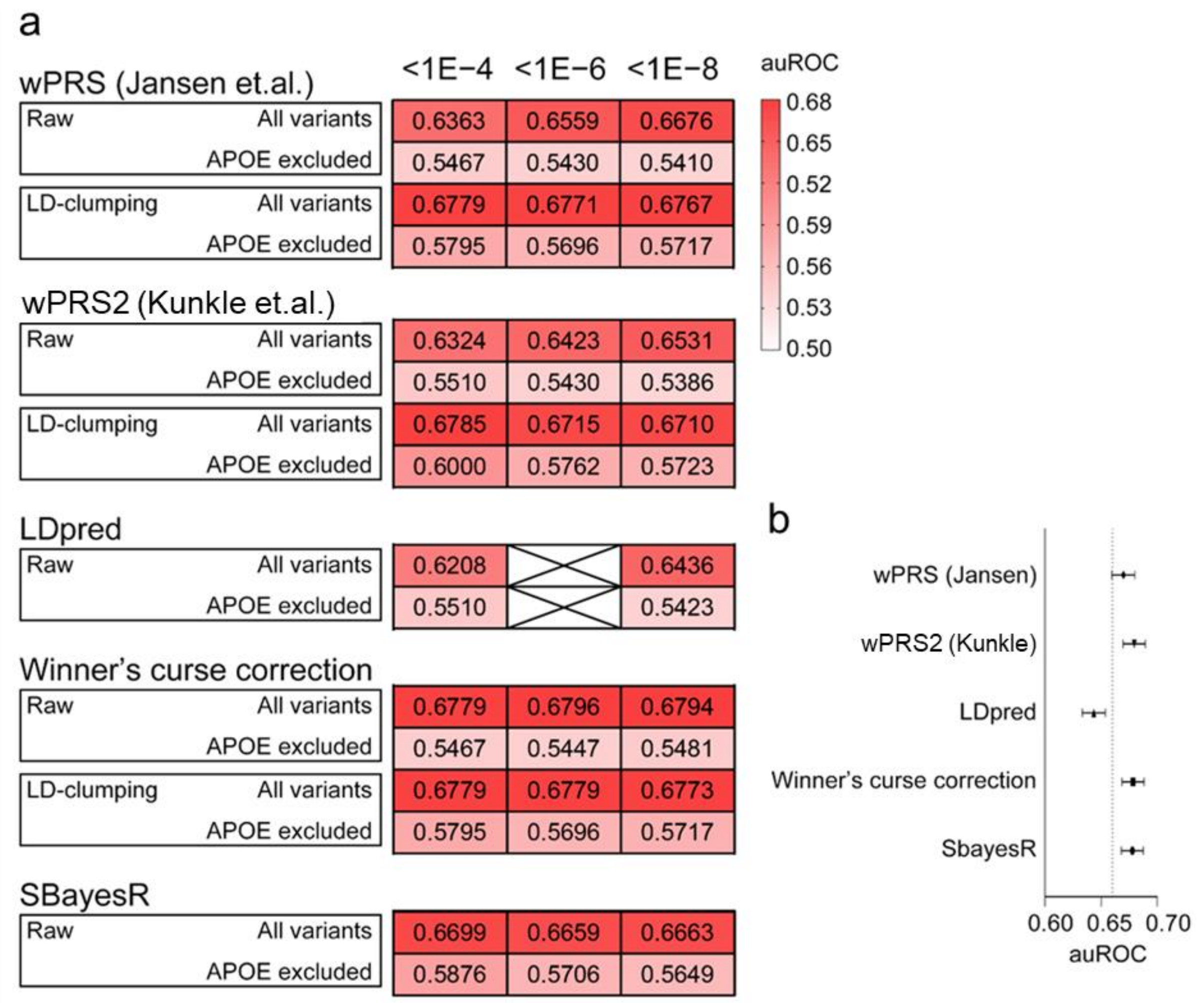

(a) Performance of the different weighted polygenic risk score models for disease classification accuracy in the European-descent cohorts using different variants sets selected by different *p*-value thresholds. (b) Comparison of the different models for disease classification accuracy. The optimal condition for each model was included for the plotting. Data are shown as means with 95% confidence intervals. auROC, area under the receiver operating characteristic curve; LD, linkage disequilibrium; wPRS, weighted polygenic risk score.

Supplementary Figure 2. Performance of different prediction models for disease classification accuracy in the European-descent cohorts without validation

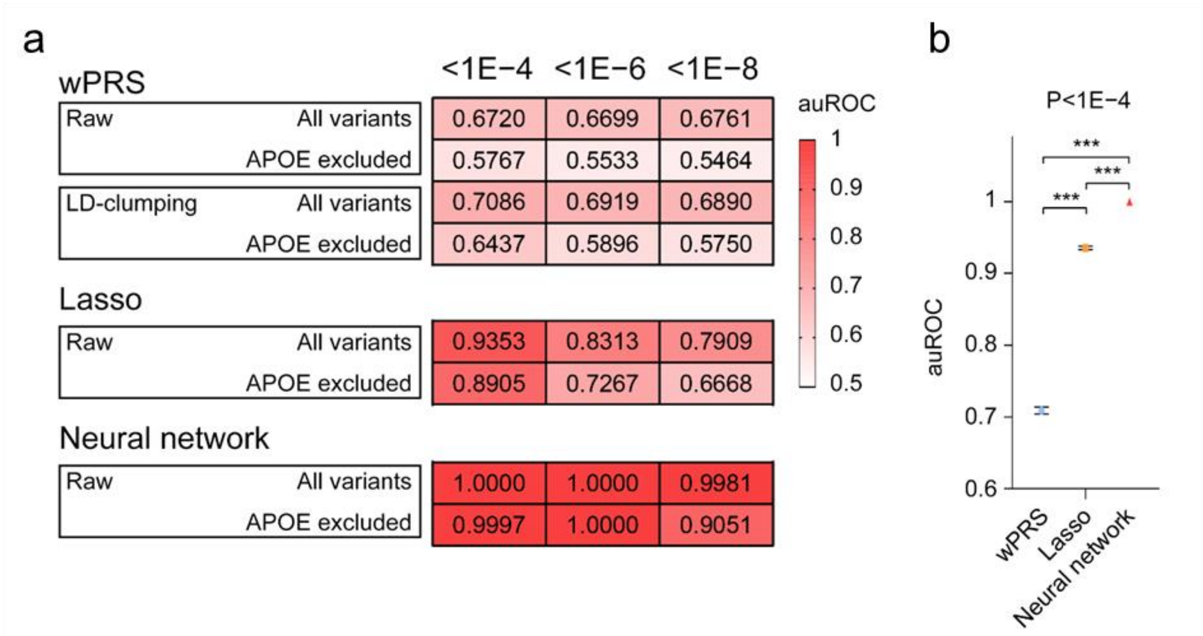

(a) Performance of the different prediction models for disease classification accuracy in the European-descent cohorts without validation. (b) Comparison of the different models for disease classification accuracy. auROC, area under the receiver operating characteristic curve; LD, linkage disequilibrium; wPRS, weighted polygenic risk score. Data are means with 95% confidence intervals. One-way ANOVA followed by Bonferroni's *post hoc* test: ****p* < 0.001. Lasso, least absolute shrinkage and selection operator; wPRS, weighted polygenic risk score.

Supplementary Figure 3. Performance of different prediction models for disease classification accuracy in the European-descent cohorts using the five-fold cross-validation method

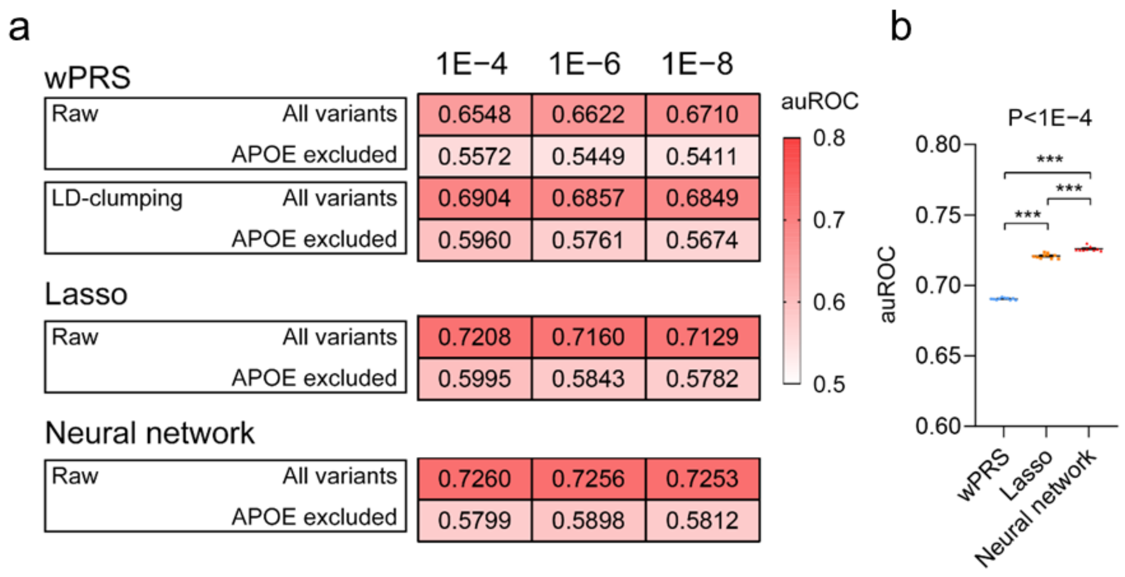

(a) Performance of the different prediction models for disease classification accuracy in the European-descent cohorts using the five-fold cross-validation method. (b) Comparison of the different models for disease classification accuracy. Data are means with 95% confidence intervals. One-way ANOVA followed by Bonferroni's *post hoc* test: ****p* < 0.001 (*n* = 10 data points per category). auROC, area under the receiver operating characteristic curve; LD, linkage disequilibrium; lasso, least absolute shrinkage and selection operator; wPRS, weighted polygenic risk score.

Supplementary Figure 4. Optimization of the neural network model for classifying Alzheimer's disease risk using an independent cohort for cross-validation

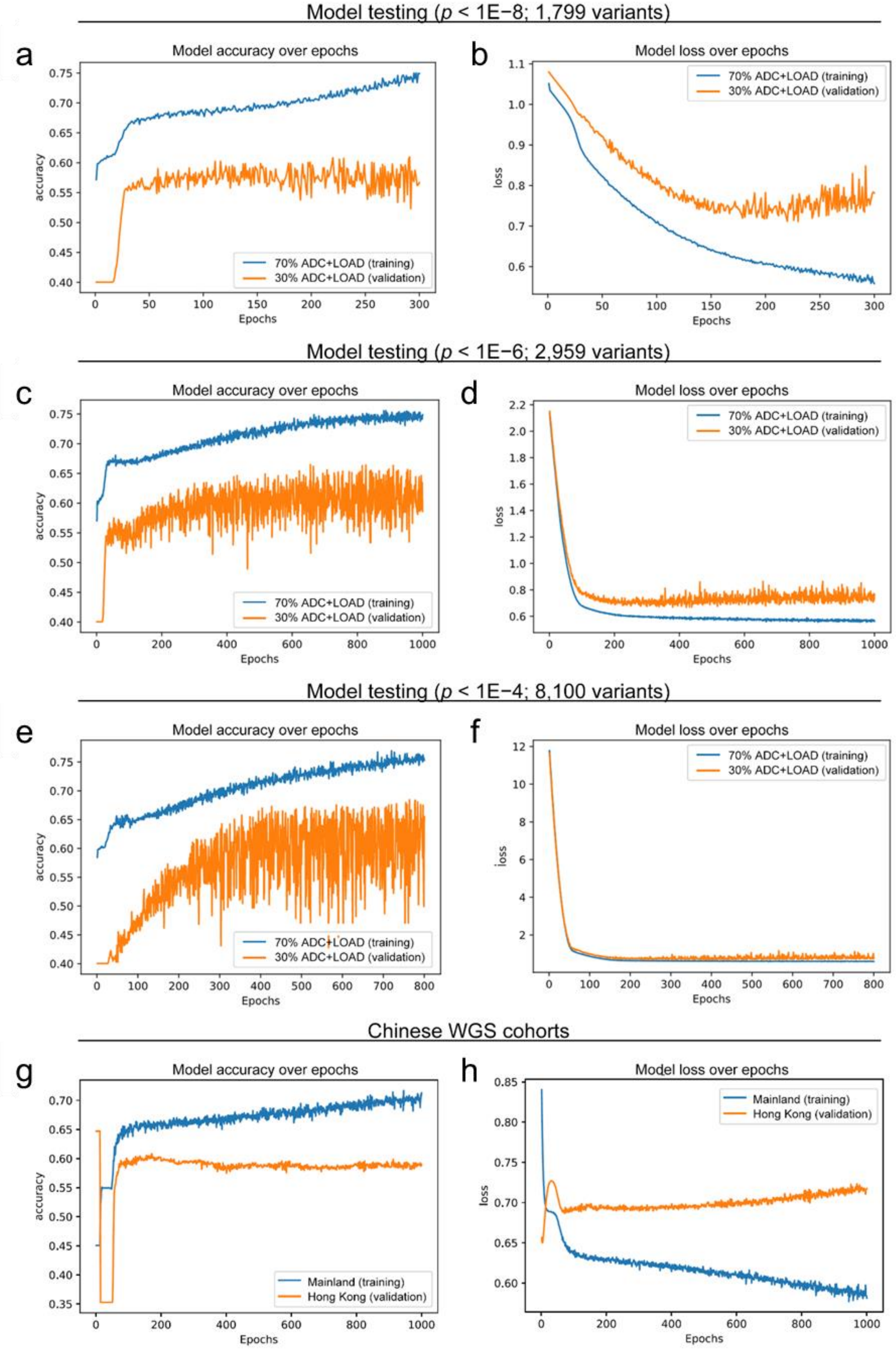

(a–h) Model performance of the neural network model during the model training. ADC, National Institute on Aging Alzheimer's Disease Centers cohort; LOAD, Late Onset Alzheimer's Disease Family Study cohort; WGS, whole-genome sequencing.

Supplementary Figure 5. Performance of different polygenic score models in the European-descent Alzheimer's disease cohorts

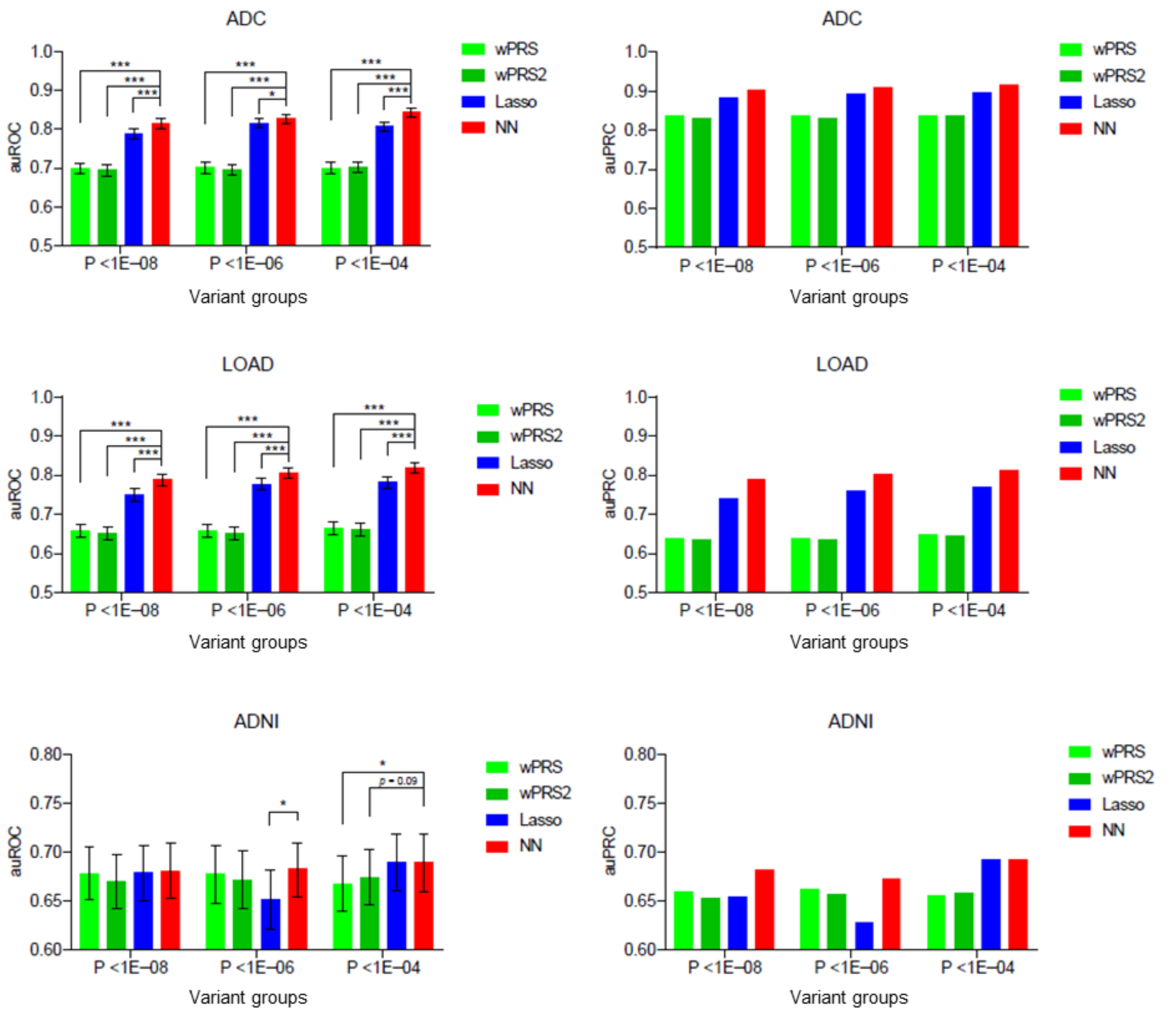

Comparisons of the auROCs and auPRCs of AD cohorts obtained by different models. For auROCs, data are shown as means with 95% confidence intervals. Bootstrap one-tailed test: *p < 0.05, **p < 0.01, ***p < 0.001. For auPRCs, data are shown as means. AD, Alzheimer's disease; ADC, National Institute on Aging Alzheimer's Disease Centers cohort; ADNI, Alzheimer's Disease Neuroimaging Initiative cohort; auPRC, area under the precision-recall curve; auROC, area under the receiver operating characteristic curve; lasso, least absolute shrinkage and selection operator; LOAD, Late Onset Alzheimer's Disease Family Study cohort; NN, neural network; wPRS, weighted polygenic risk score using results from Jansen's summary statistics; wPRS2, a parallel weighted polygenic risk score analysis using results from IGAP 2019 summary statistics.

Supplementary Figure 6. Performance of different prediction models for disease classification accuracy in the European-descent cohorts stratified by ethnic group

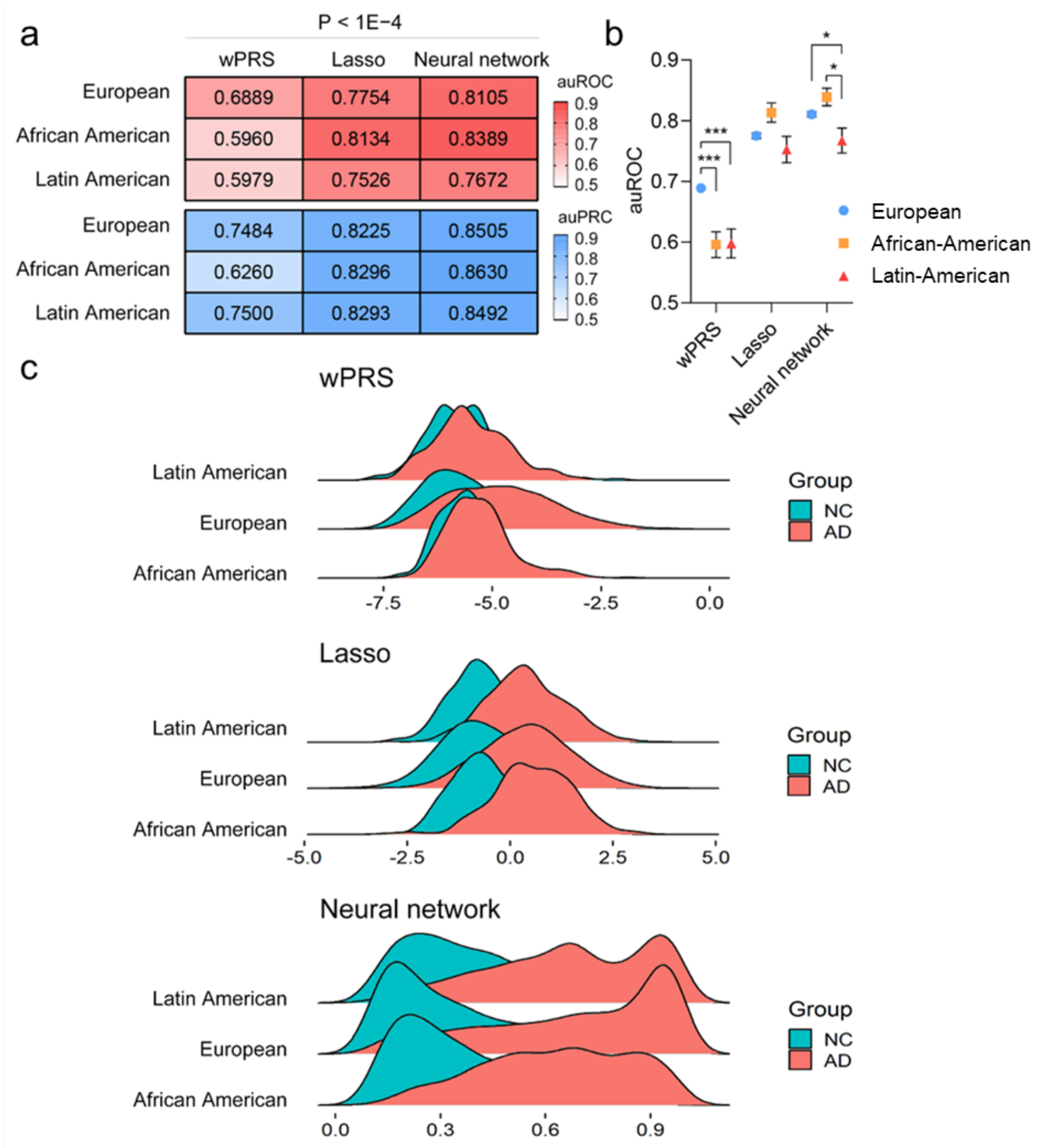

(a) Performance of the different prediction models for disease classification accuracy in the European-descent cohorts stratified by ethnic group. (b) Comparison of the different models for disease classification accuracy. Data are means with 95% confidence intervals. One-way ANOVA followed by Bonferroni's *post hoc* test: **p* < 0.05, ****p* < 0.001. (c) Visualization of the polygenic risk score distribution stratified by phenotype and ethnic group. AD, Alzheimer's disease; auPRC, area under the precision-recall curve; auROC, area under the receiver operating characteristic curve; lasso, least absolute shrinkage and selection operator; NC, normal control; wPRS, weighted polygenic risk score.

Supplementary Figure 7. Performance of different prediction models for disease classification accuracy in the European-descent population stratified by sex

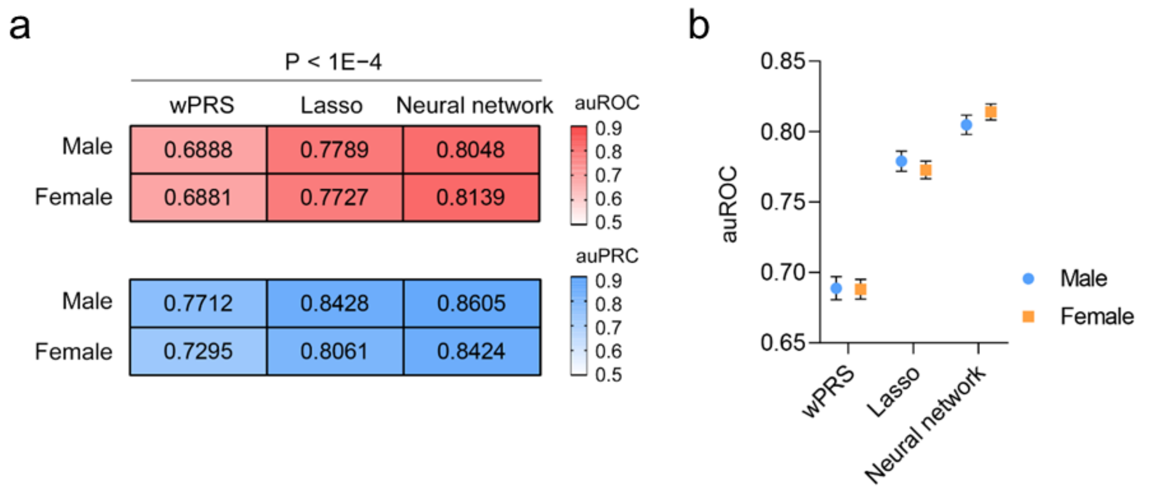

(a) Performance of the different prediction models for disease classification accuracy in the European-descent population stratified by sex. (b) Comparison of the different models for disease classification accuracy, stratified by sex. Data are means with 95% confidence intervals. auPRC, area under the precision-recall curve; auROC, area under the receiver operating characteristic curve; lasso, least absolute shrinkage and selection operator; wPRS, weighted polygenic risk score.

Supplementary Figure 8. Performance of different prediction models for disease classification accuracy in the European-descent population stratified by age group

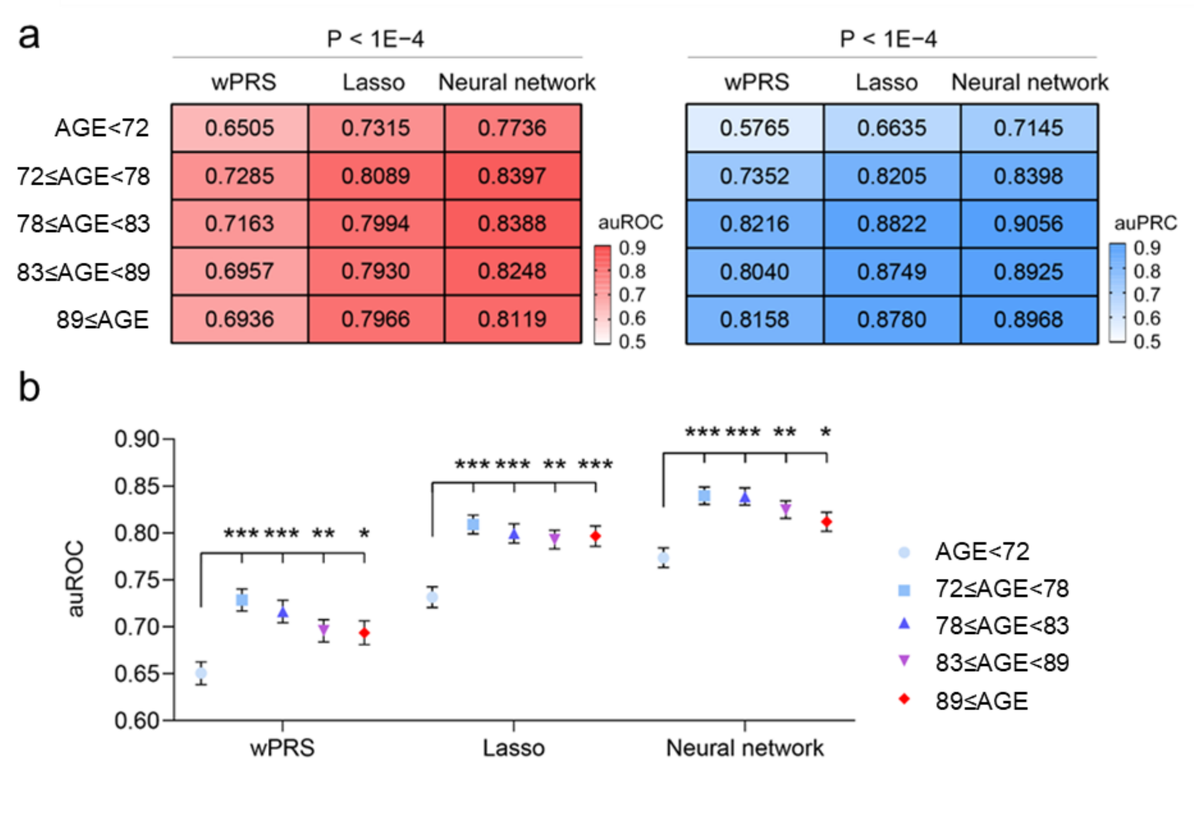

(a) Performance of the different prediction models for disease classification accuracy in the European-descent population stratified by age group. (b) Comparison of the different models for disease classification accuracy, stratified by age group. Data are means with 95% confidence intervals. One-way ANOVA followed by Bonferroni's *post hoc* test: *$p < 0.05$, **$p < 0.01$, ***$p < 0.001$. auPRC, area under the precision-recall curve; auROC, area under the receiver operating characteristic curve; lasso, least absolute shrinkage and selection operator; wPRS, weighted polygenic risk score.

Supplementary Figure 9. Performance of trans-ethnic prediction models for disease classification accuracy

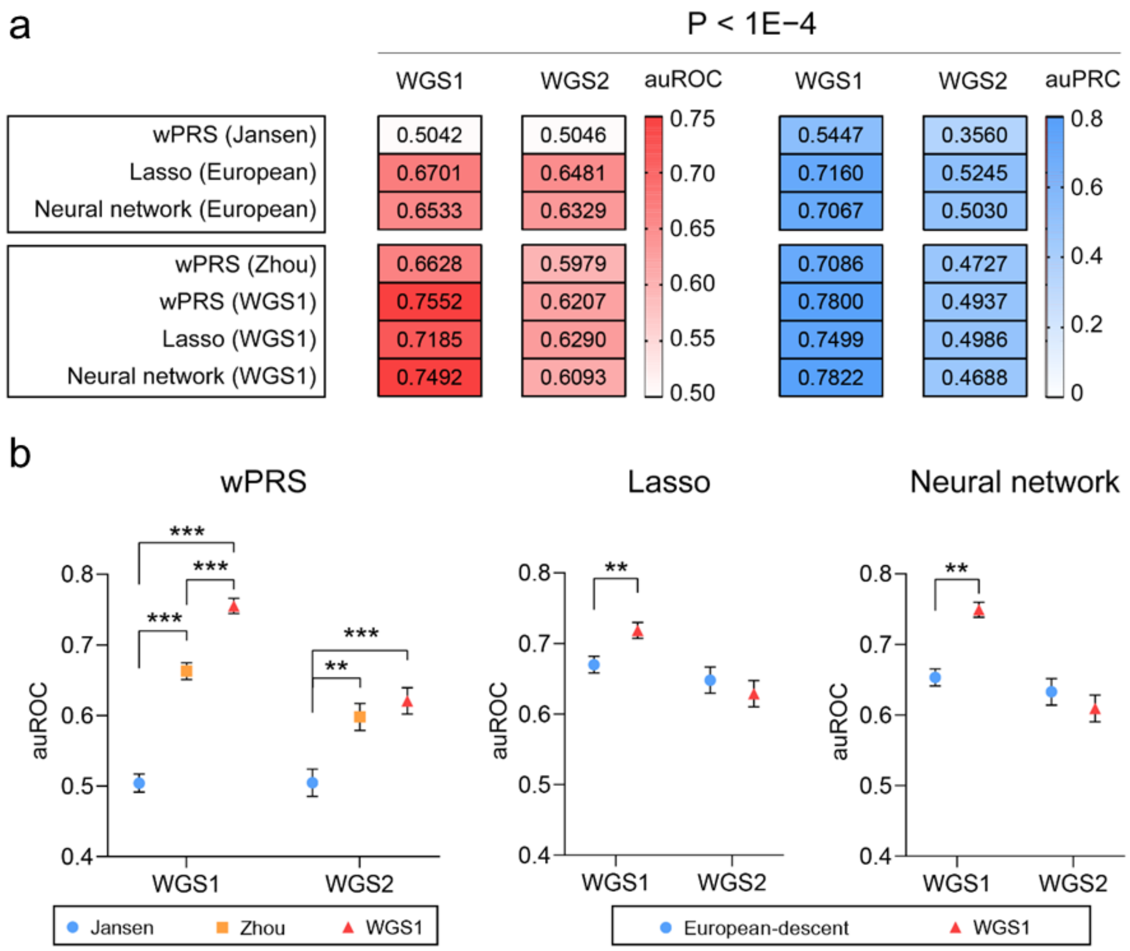

(a) Performance of different trans-ethnic prediction models for disease classification accuracy. (b) Comparison of the different models for disease classification accuracy. Colors in left panel denote the different GWAS summary statistics used to construct the models: Jansen et al. (blue), Zhou et al. (orange), and results from WGS1 cohort (red). Colors in right panel denote different reference datasets used to construct the models: European-descent datasets (i.e. the ADC, LOAD, and ADNI datasets; blue), and WGS1 cohort (red). Data are means with 95% confidence intervals. One-way ANOVA followed by Bonferroni's *post hoc* test: ***p* < 0.01, ****p* < 0.001. ADC, National Institute on Aging Alzheimer's Disease Centers cohort; ADNI, Alzheimer's Disease Neuroimaging Initiative cohort; auPRC, area under the precision-recall curve; auROC, area under the receiver operating characteristic curve; lasso, least absolute shrinkage and selection operator; LOAD, Late Onset Alzheimer's Disease Family Study cohort; *p*, *p*-value; WGS, whole-genome sequencing; WGS, whole-genome sequencing; WGS1, Chinese WGS cohort 1; WGS2, Chinese WGS cohort 2; wPRS, weighted polygenic risk score.

Supplementary Figure 10. Genomic correlations among the polygenic risk scores obtained from the trans-ethnic prediction models in Chinese WGS cohort 1

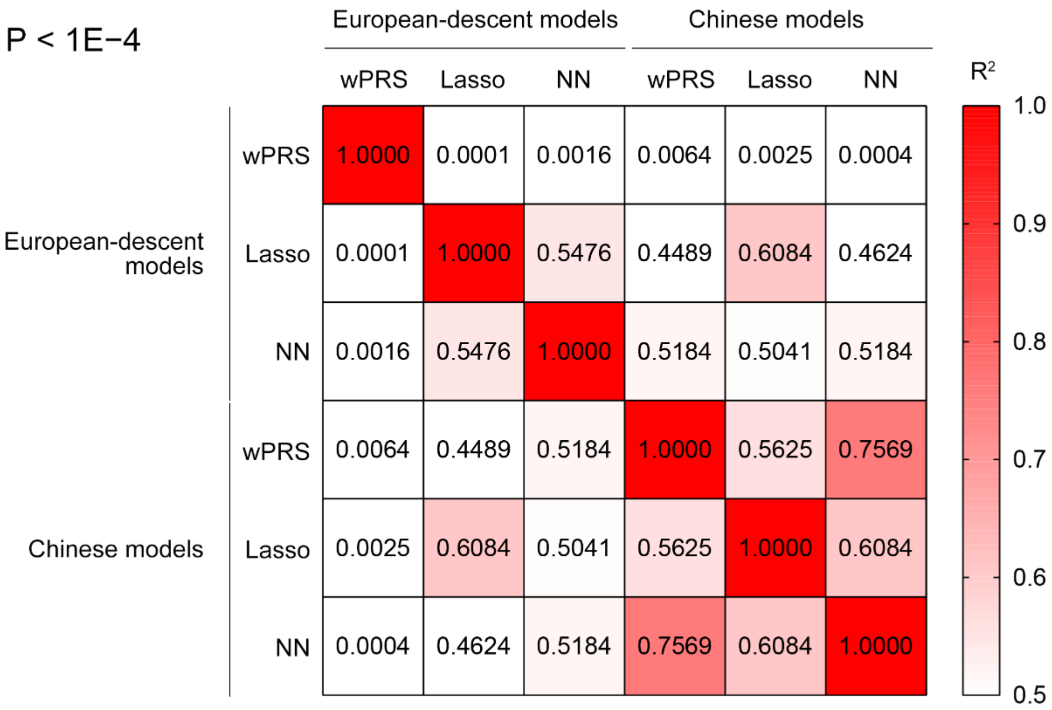

R^2 was calculated using Spearman's rank correlation test. lasso, least absolute shrinkage and selection operator; NN, neural network; *p*, *p*-value; WGS, whole-genome sequencing; WGS1, Chinese WGS cohort 1; wPRS, weighted polygenic risk score.

Supplementary Figure 11. Performance of polygenic risk models for classifying Alzheimer's disease risk in the Chinese Alzheimer's disease whole-genome sequencing cohorts

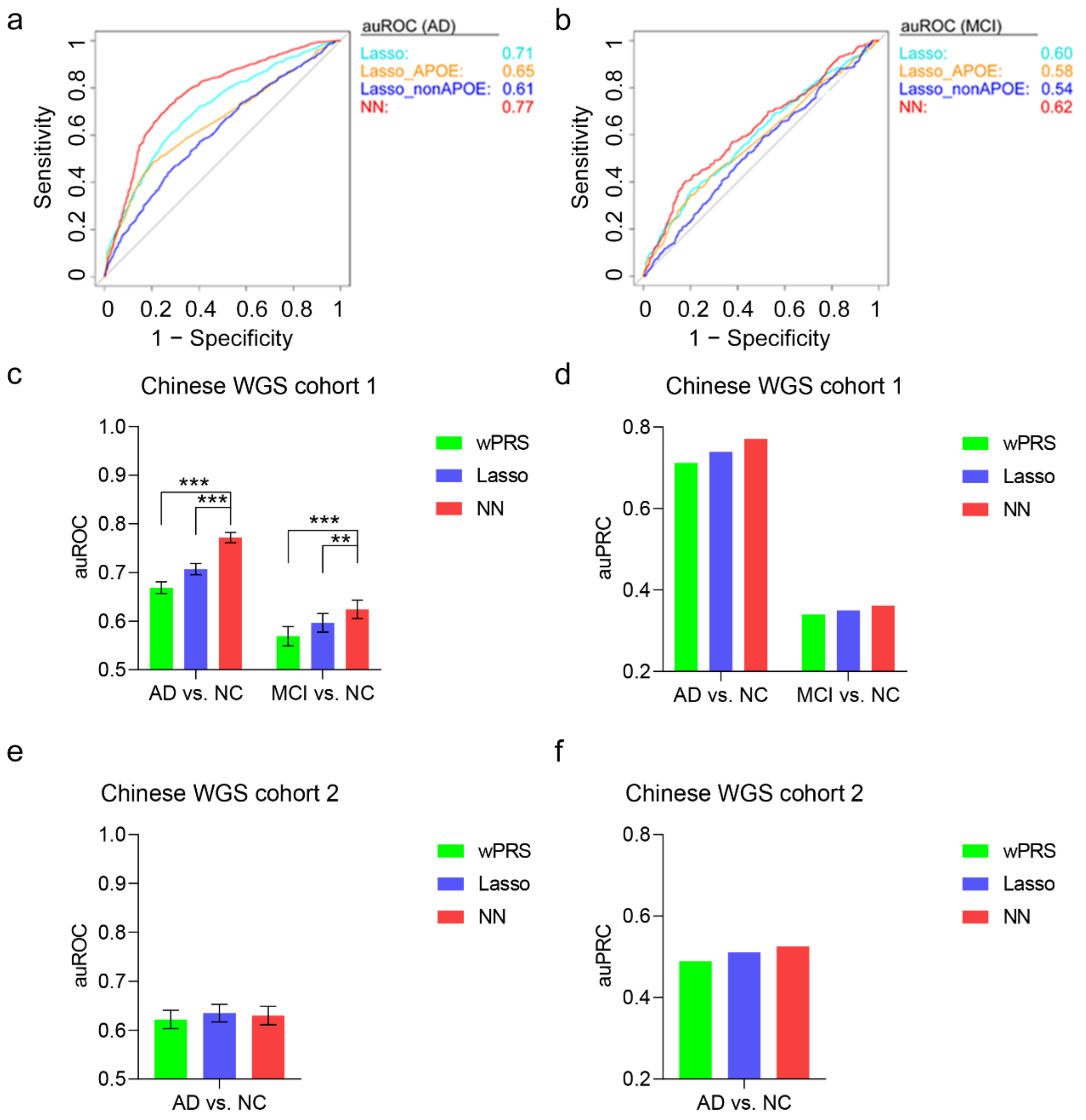

(a–b) ROC curves for model performance in classifying (a) AD and (b) MCI patients in Chinese WGS cohort 1. (c–f) Model performance for classifying AD and MCI patients in Chinese WGS cohorts 1 and 2. For auROC, the y-axis shows the mean auROC, with error bars representing 95% confidence intervals. The data were analyzed using a bootstrap two-tailed test: *$p < 0.1$, **$p < 0.01$, ***$p < 0.001$. For auPRC, y-axis shows the mean auPRC. AD, Alzheimer's disease; auPRC, area under the precision-recall curve; auROC, area under the receiver operating characteristic curve; lasso, least absolute shrinkage and selection operator; Lasso_APOE, lasso model constructed using variants in *APOE* regions; lasso_nonAPOE, lasso model constructed using variants outside of *APOE* regions; MCI, mild cognitive impairment; NC, normal control; NN, neural network; WGS, whole-genome sequencing; WGS1, Chinese WGS cohort 1; WGS2, Chinese WGS cohort 2; wPRS, weighted polygenic risk score.

Supplementary Figure 12. Comparison of the classification accuracy of modified polygenic risk score models using 37 variants in the Chinese population

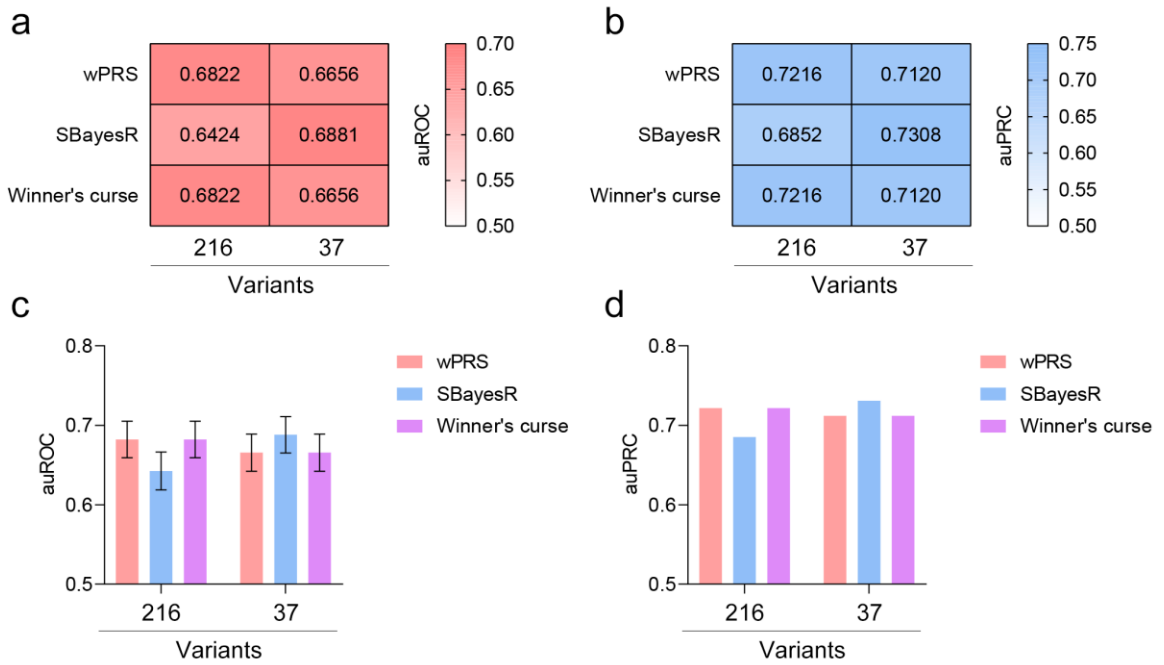

(a, b) Performance of the different weighted polygenic risk score models for disease classification accuracy in the Chinese population using different variants sets measured by (a) auROC and (b) auPRC. (c, d) Comparison of disease classification accuracy between different models. (c) auROC and (d) auPRC values are plotted as means, with error bars denoting 95% confidence intervals. The numbers of variants are marked underneath each plot (both heatmaps and bar charts). auPRC, area under the precision-recall curve; auROC, area under the receiver operating characteristic curve; wPRS, weighted polygenic risk score.

Supplementary Figure 13. Evaluation of different prediction models using 37 variants for disease classification accuracy in European-descent cohorts and Chinese WGS cohort 1 using the five-fold cross-validation method

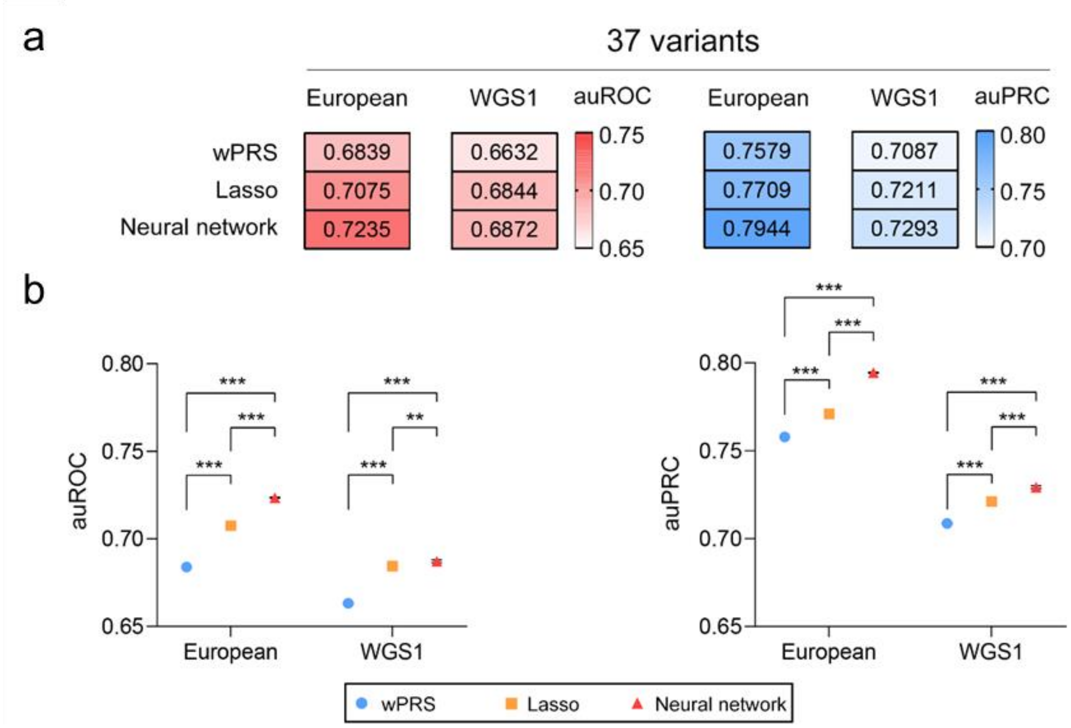

(a) Performance of the different prediction models using 37 variants for disease classification accuracy in European-descent cohorts and the WGS1 dataset using the five-fold cross-validation method. (b) Comparison of the different models for disease classification accuracy. Data are means with 95% confidence intervals. One-way ANOVA followed by Bonferroni's *post hoc* test: **$p < 0.01$, ***$p < 0.001$ ($n = 10$ data points per category). auPRC, area under the precision-recall curve; auROC, area under the receiver operating characteristic curve; lasso, least absolute shrinkage and selection operator; WGS, whole-genome sequencing; WGS1, Chinese WGS cohort 1; wPRS, weighted polygenic risk score.

Supplementary Figure 14. Comparison between models using 37 variants and variants selected by *p*-value thresholds for classifying Alzheimer's disease risk in the European-descent cohorts using the five-fold cross-validation method

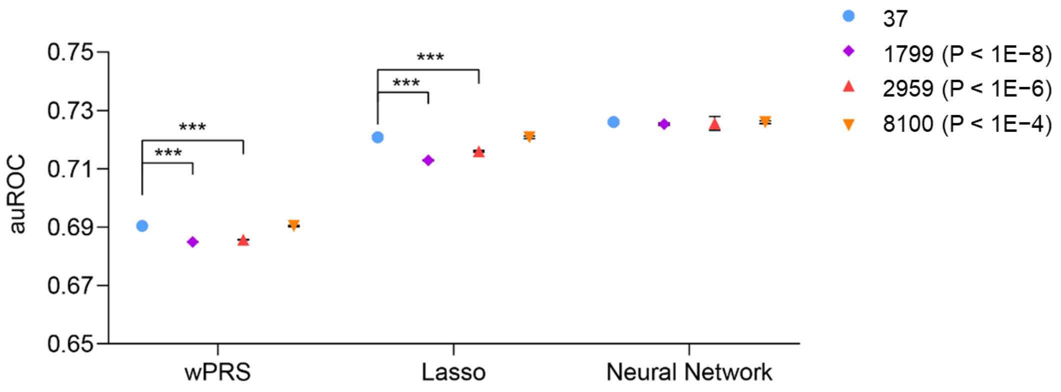

Data are means with 95% confidence intervals. One-way ANOVA followed by Bonferroni's *post hoc* test: ***$p < 0.001$ ($n = 10$ data points per category). auROC, area under the receiver operating characteristic curve; *p*, *p*-value; wPRS, weighted polygenic risk score.

Supplementary Figure 15. Comparison between models using 37 variants and variants selected by *p*-value thresholds for classifying Alzheimer's disease risk in the Chinese population

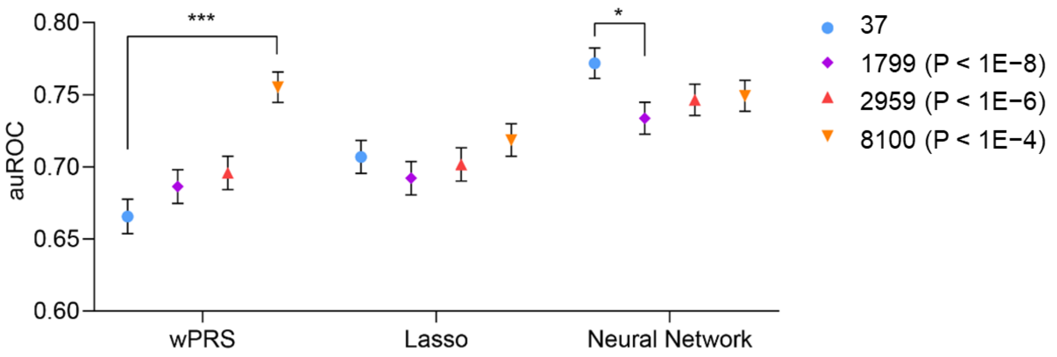

Data are means with 95% confidence interval. Two-way ANOVA followed by Benjamini--Hochberg's *post hoc* test comparing results from 37 variants and other groups: **p* < 0.05, ****p* < 0.001. auROC, area under the receiver operating characteristic curve; lasso, least absolute shrinkage and selection operator; *p*, *p*-value; wPRS, weighted polygenic risk score.

Supplementary Figure 16. Performance of trans-ethnic prediction models using 37 variants for disease classification accuracy in the European-descent cohorts and WGS1 dataset

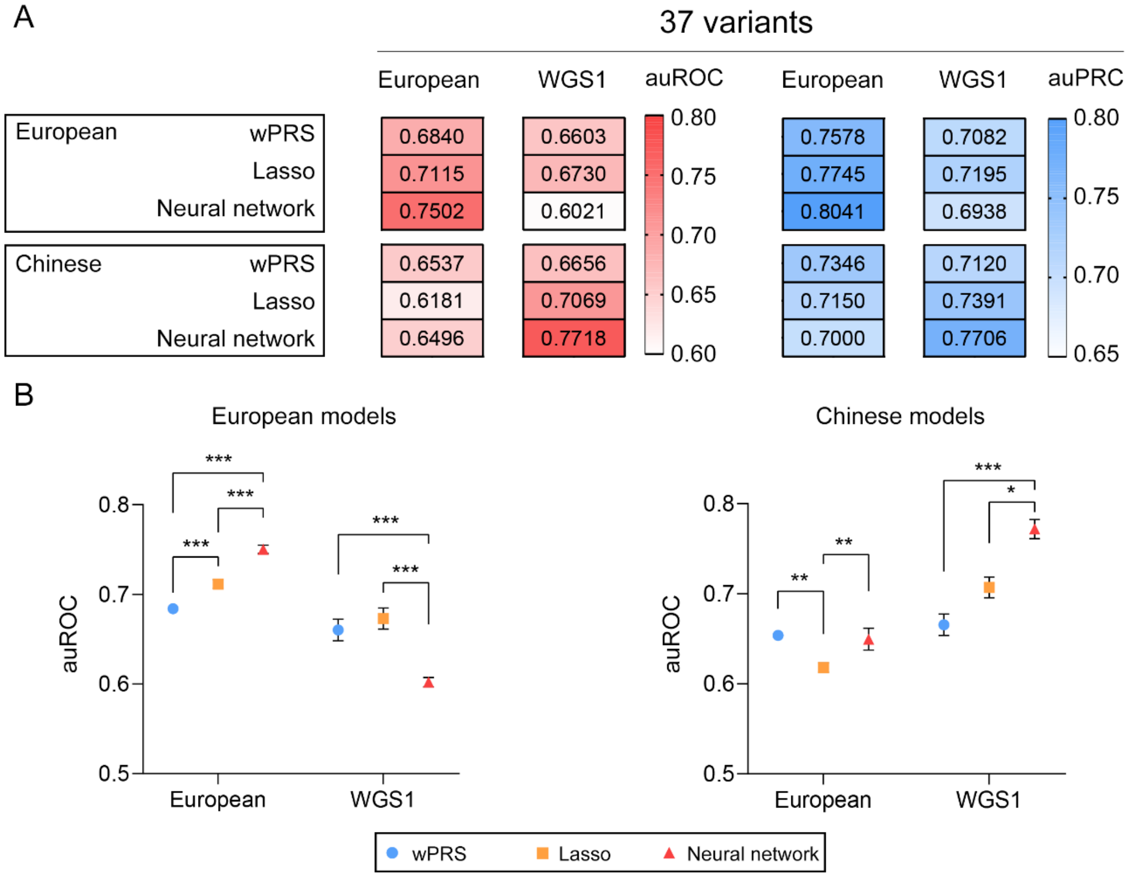

(a) Performance of trans-ethnic prediction models using 37 variants for disease classification accuracy in the European-descent cohorts and WGS1 dataset. (b) Comparison of the different models for disease classification accuracy in different ethnic groups. Data are means with 95% confidence intervals. One-way ANOVA followed by Bonferroni's *post hoc* test: *$p < 0.05$, **$p < 0.01$, ***$p < 0.001$. auPRC, area under the precision-recall curve; auROC, area under the receiver operating characteristic curve; lasso, least absolute shrinkage and selection operator; WGS, whole-genome sequencing; WGS1, Chinese WGS cohort 1; WGS2, Chinese WGS cohort 2; wPRS, weighted polygenic risk score.

Supplementary Figure 17. Classification of Alzheimer's disease in the Chinese population using neural network models with different variant sets

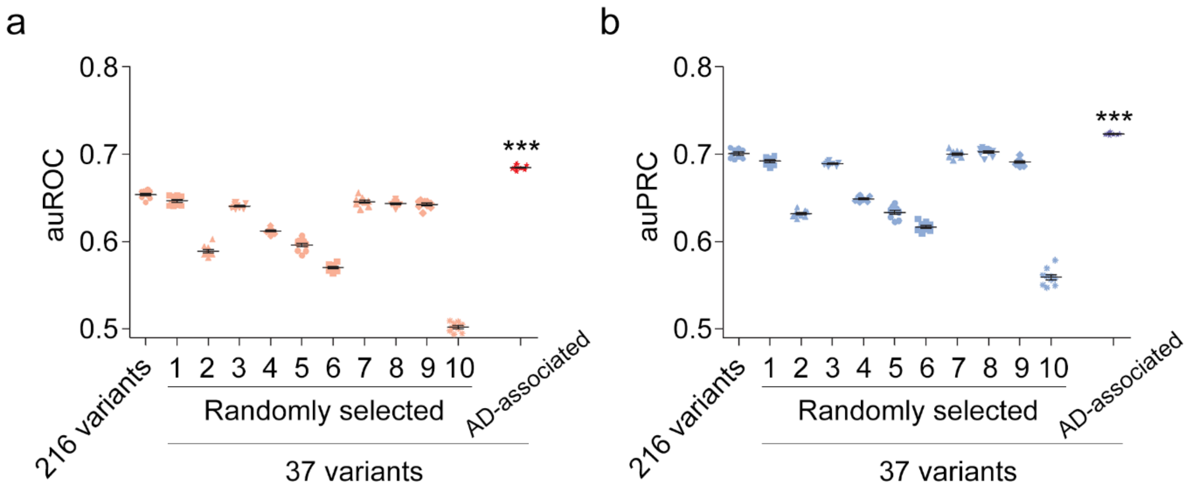

Dot plots show the classification accuracy of neural network models constructed with five-fold cross-validation based on the following: all 216 AD variants reported by genome-wide association studies, 10 sets of 37 variants randomly selected from those 216 variants ("Randomly selected"), and the 37 AD variants that showed significant associations in the Chinese population ("AD-associated"). Data are means ± SEM. One-way ANOVA followed by Tukey's *post hoc* test comparing (a) auROC and (b) auPRC with all other variants groups: ***$p < 0.001$. AD, Alzheimer's disease; auPRC, area under the precision-recall curve; auROC, area under the receiver characteristics curve; SEM, standard error of the mean.

Supplementary Figure 18. Performance of polygenic risk models for classifying Alzheimer's disease risk in Chinese WGS cohort 2

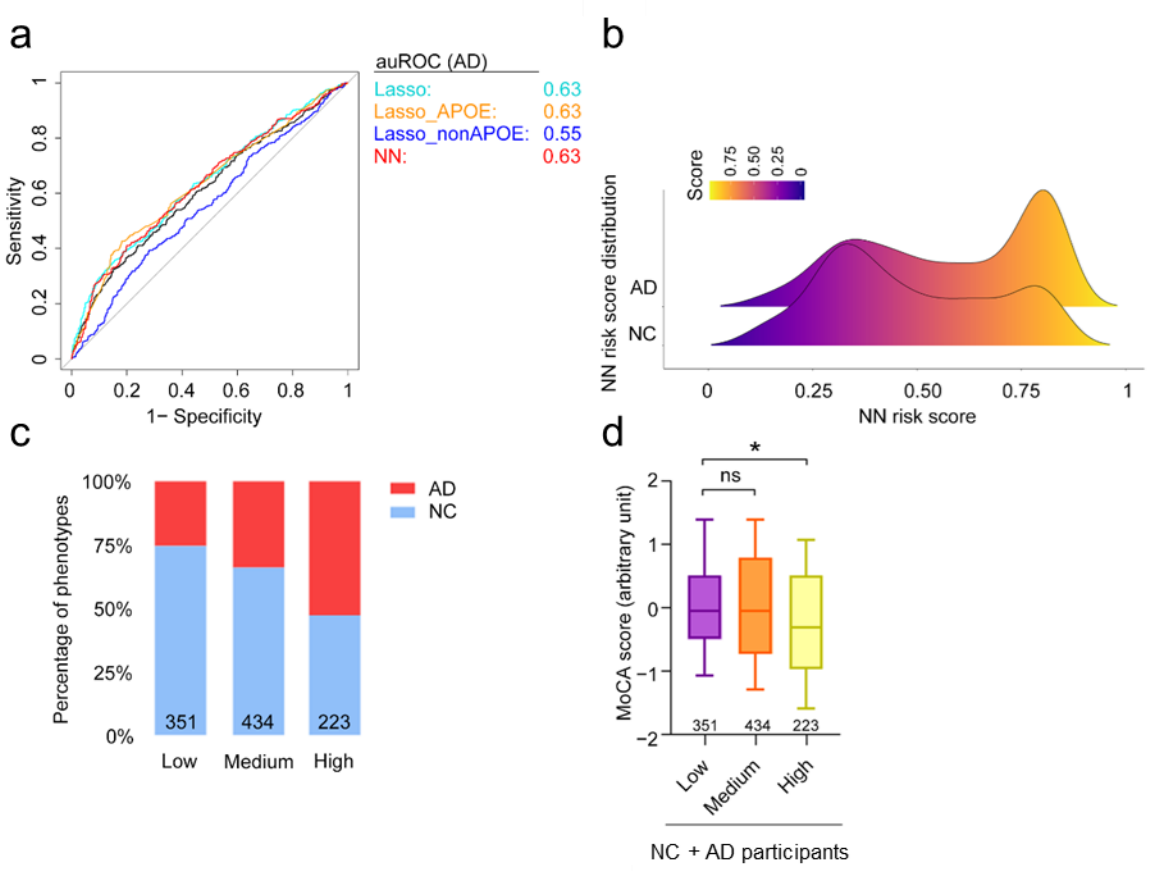

(a) ROC curves of the polygenic score classification of patients with AD in Chinese WGS cohort 2. (b) Distribution of polygenic risk scores derived from the NN model for each phenotype group. (c) Percentages of each phenotype group in the low-, medium-, and high-risk groups. (d) Associations between polygenic risk score and MoCA score in NCs and patients with AD. Data are presented as box-and-whisker plots; boxes indicate the 25th to 75th percentiles, and whiskers indicate the 10th and 90th percentiles. The numbers of individuals in the corresponding groups are shown at the bottom of each plot. **p* < 0.05; robust linear regression model. AD, Alzheimer's disease; auROC, area under the receiver operating characteristic curve; lasso, least absolute shrinkage and selection operator; Lasso_APOE, lasso model constructed using variants in *APOE* regions; lasso_nonAPOE, lasso model constructed using variants outside of *APOE* regions; MoCA, Montreal Cognitive Assessment; NC, normal control; NN, neural network; ns, not significant; WGS, whole-genome sequencing; wPRS, weighted polygenic risk score.

Supplementary Figure 19. Associations between polygenic risk score and cognitive performance in patients with mild cognitive impairment

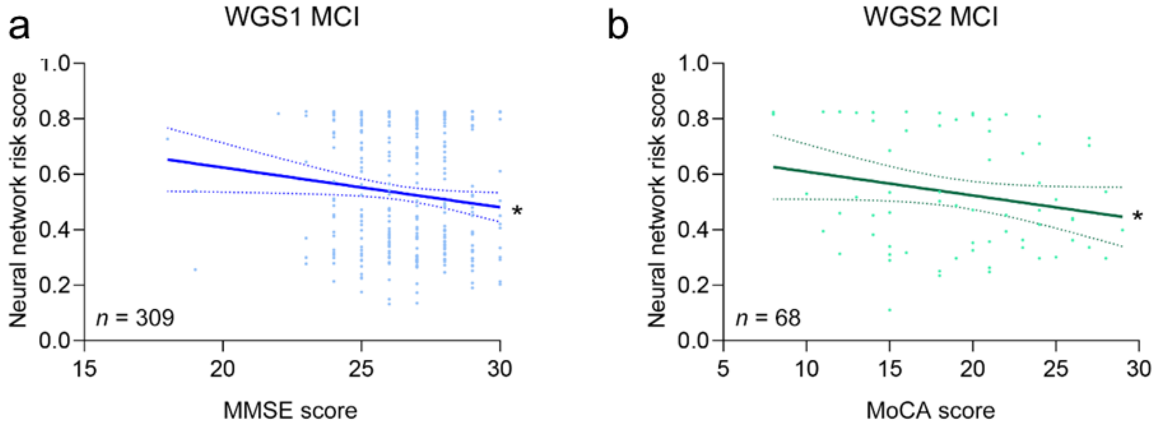

(a) Association results in individuals with MCI from the WGS1 dataset. (b) Association results in patients with MCI from the WGS2 dataset. *$p < 0.05$, robust regression controlling for age, sex, and top-five genetic principal components. MCI, mild cognitive impairment; MMSE, Mini–Mental State Exam; MoCA, Montreal Cognitive Assessment; n, number of samples; WGS, whole-genome sequencing; WGS1, Chinese WGS cohort 1; WGS2, Chinese WGS cohort 2.

Supplementary Figure 20. Associations between polygenic scores and brain region volumes

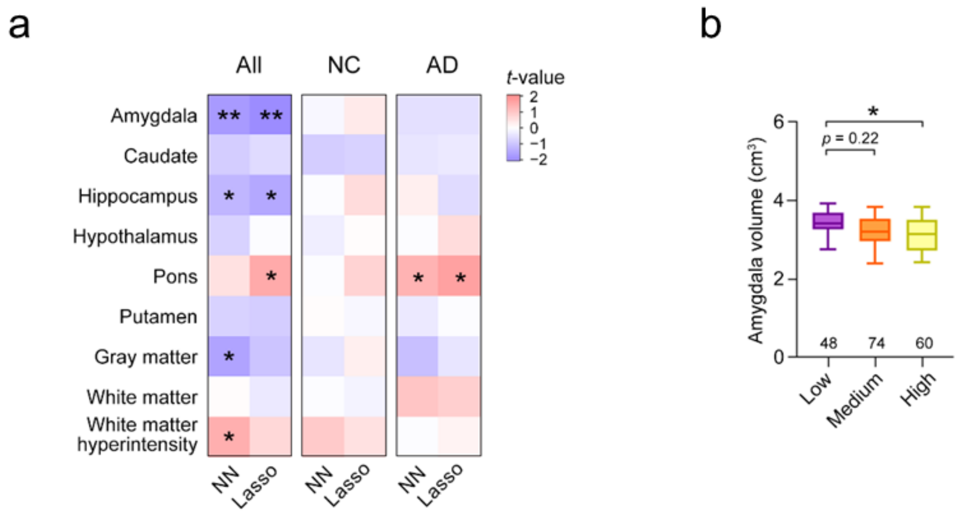

(a) Associations between polygenic score obtained from neural network model and volumes of specific brain regions. Robust linear regression: *$p < 0.05$, **$p < 0.01$. (b) Associations between polygenic score obtained from neural network models and amygdala volume. Data are presented as box-and-whisker plots; boxes indicate the 25th to 75th percentiles, and whiskers indicate the 10th and 90th percentiles. The numbers of individuals in the corresponding group are shown at the bottom of each plot. *$p < 0.05$, robust linear regression model. AD, Alzheimer's disease; NC, normal control; NN, neural network.

Supplementary Figure 21. Effects of node numbers in the penultimate layer of the neural network model on the biomarker association analysis

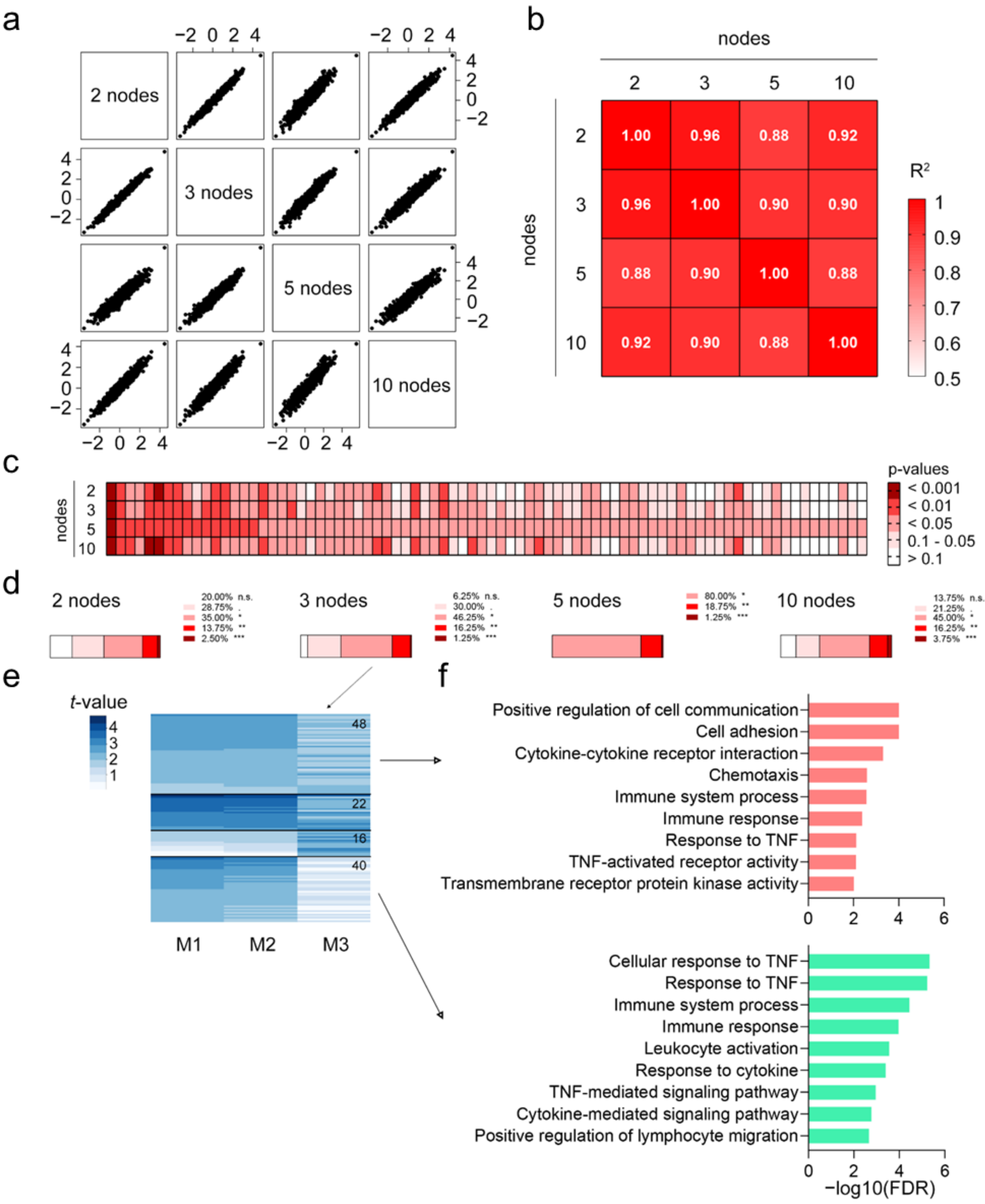

(a) Scatter plots showing polygenic risk scores were obtained from neural network models with different numbers of nodes in the penultimate layer. (b) Spearman's rank correlations among the polygenic risk scores. (c) Association *p*-values between the polygenic risk scores and plasma protein levels among proteins showing an association in the five-node model. (d) Summary of the *p*-values obtained from the association analysis between the polygenic risk scores and plasma protein levels. n.s., for $p > 0.1$, $\cdot p = 0.1$–0.05, $*p < 0.05$, $**p < 0.01$, $***p <$

0.001. (e) Heatmap of proteins clustered by the *k*-means clustering method according to their associations (*t*-values) between plasma protein levels and subscores from the models with three nodes in the penultimate layer. Numbers denote the number of proteins in each cluster. (f) Pathway and Gene Ontology enrichment analyses of plasma proteins in the first and last clusters. FDR, false discovery rate; R^2, correlation coefficient; TNF, tumor necrosis factor.

Supplementary Figure 22. Interpretation of the polygenic risk effects on the modulation of gene expression

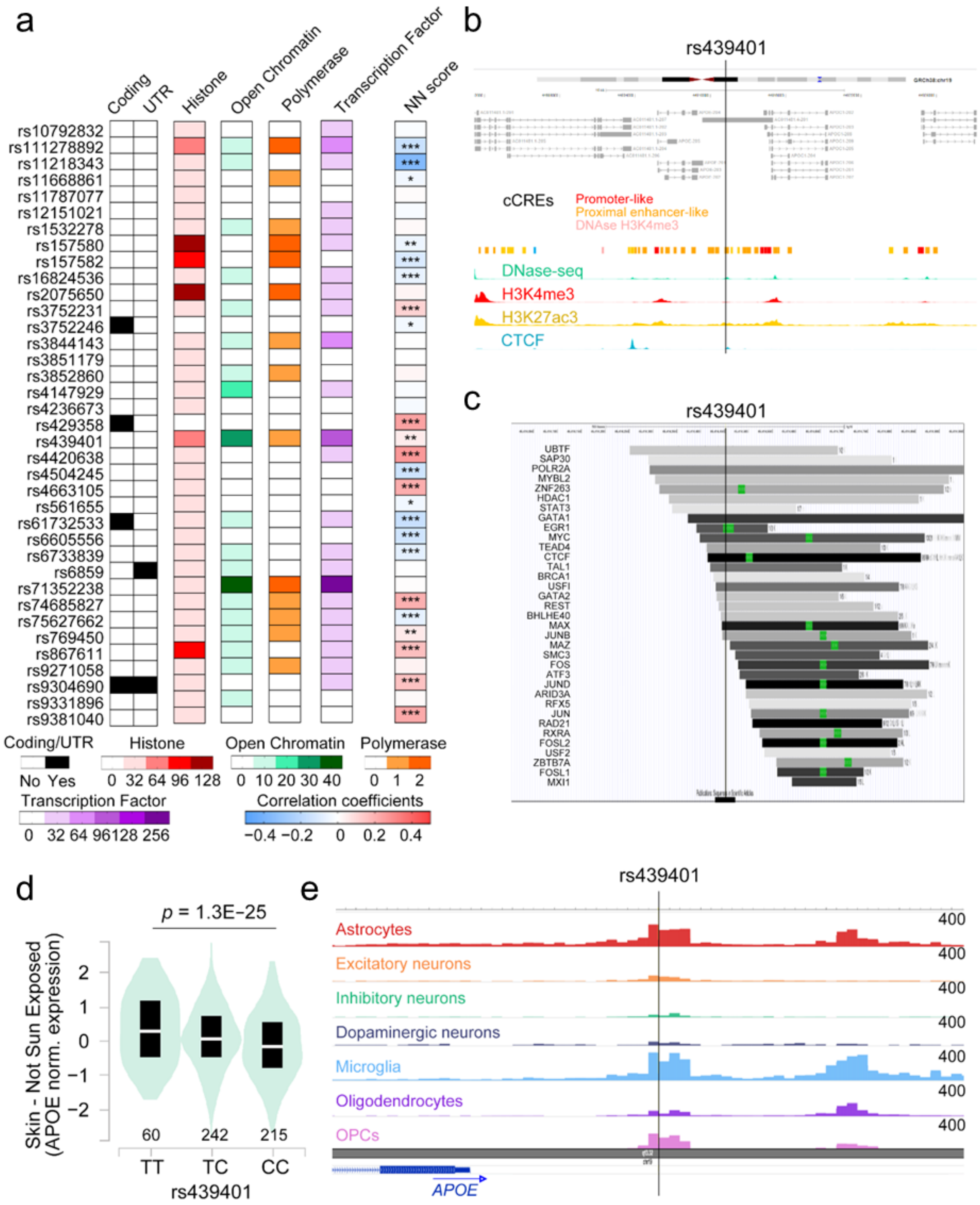

(a) Summary of the properties of the 37 variants used for polygenic risk analysis. The annotation on coding and UTR (i.e. yes or no), histone modification, open chromatin, polymerase, and transcription factors (i.e. number of records in the ENCODE Screen database) were obtained from SNPnexus (https://www.snp-nexus.org/), with the last column showing the partial correlation results between individual variants and polygenic risk scores obtained from the NN models. (b) Variant rs439401 resides in the regulatory region. The plot was obtained

from the ENCODE Screen database (https://screen.encodeproject.org/). (c) Variant rs439401 resides in the transcription factor-binding regions as annotated by the ENCODE transcription factor-binding track and visualized in the UCSC Genome Browser (https://genome.ucsc.edu/). (d) Variant rs439401 is associated with APOE expression in skin tissue. Data were obtained from the GTEx database (https://gtexportal.org/home/). (e) Variant rs439401 is in the chromatin accessible regions as annotated from single-cell ATAC-seq data (PMID: 33106633)[7]. ATAQ-seq, Assay for Transposase-Accessible Chromatin using sequencing; cCREs, candidate cis-regulatory elements; CTCF, CCCTC-binding factor; DNA-seq, DNase sequencing; NN, neural network; OPCs, oligodendrocyte progenitor cells; UCSC, University of California Santa Cruz; UTR, untranslated region.

Supplementary Figure 23. Design and performance of the graph neural network for disease risk classification

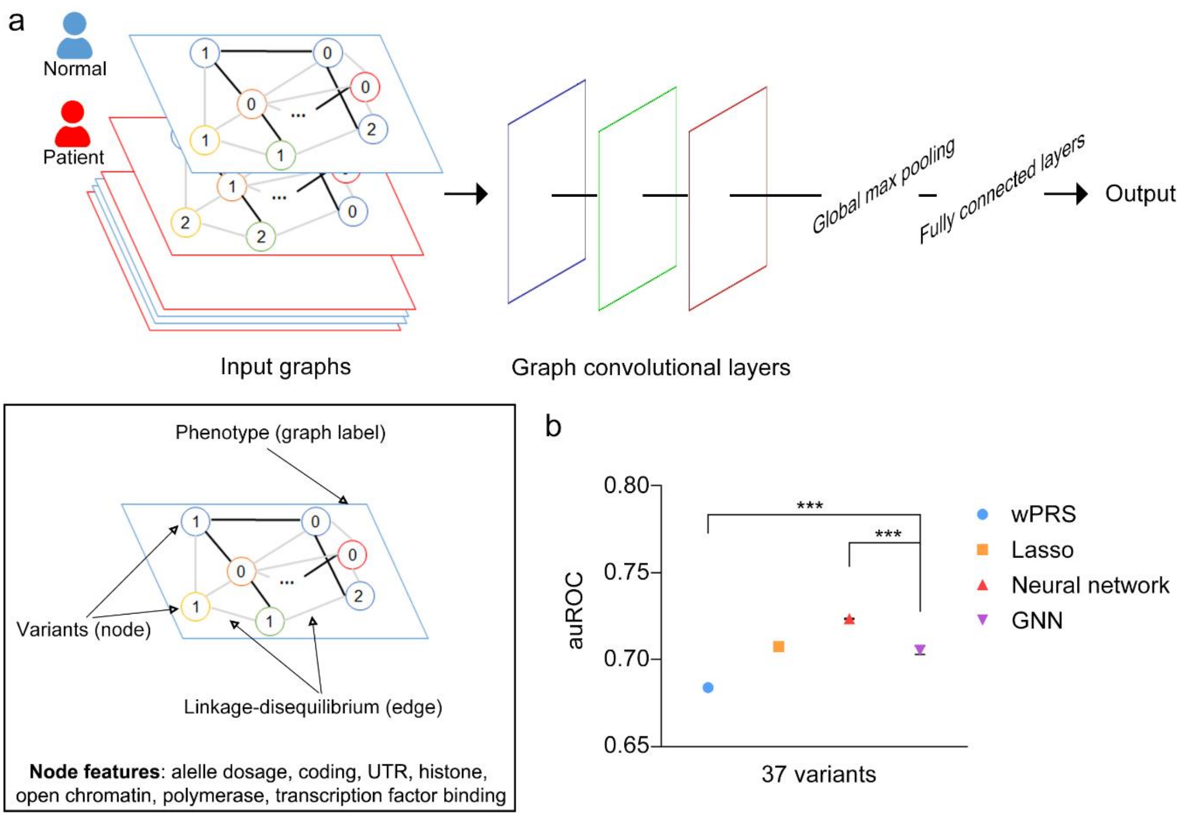

(a) Design of the graph neural network model. (b) Comparison of different models with respect to disease classification accuracy. Data are means with 95% confidence intervals. One-way ANOVA followed by Bonferroni's *post hoc* test: ***p < 0.001. auROC, area under the receiver operating characteristic curve; lasso, least absolute shrinkage and selection operator; GNN, graph neural network; UTR, untranslated region; wPRS, weighted polygenic risk score.

Supplementary Table 1. Demographic and clinical characteristics of the study cohorts (for Figure 1)

**Cohorts for polygenic score model testing (*N* = 11,352)**			
ADC (*N* = 5,692)			
	AD	NC	
Number of participants	3,946	1,746	
Mean age (SD)	79.72 (7.67)	75.51 (9.43)	
Female (%)	2,145 (54.36%)	1,138 (65.18%)	
LOAD (*N* = 4,278)			
	AD	NC	
Number of participants	2,046	2,232	
Mean age (SD)	89.15 (8.32)	80.67 (10.75)	
Female (%)	1,372 (67.06%)	1,361 (60.98%)	
ADNI (*N* = 1,382)			
	AD	NC	
Number of participants	689	693	
Mean age (SD)	77.65 (7.80)	75.29 (7.43)	
Female (%)	291 (42.24%)	387 (55.84%)	
**Chinese WGS cohorts (*N* = 3,417)**			
Chinese AD WGS cohort 1 (*N* = 2,340)			
	AD	NC	MCI
Number of participants	1,116	915	309
Mean age (SD)	67.22 (9.68)	67.44 (8.80)	69.07 (8.07)
Female (%)	629 (56.36%)	489 (53.44%)	149 (48.22%)
Mean MMSE score (SD)	14.32 (6.25)	28.31 (2.20)	26.46 (1.90)
Mean years of education (SD)	8.47 (4.90)	12.76 (3.08)	11.85 (3.46)
APOE-ε4 carriers (Allele frequency)	471 (25.40%)	157 (9.23%)	98 (18.45%)
APOE-ε2 carriers (Allele frequency)	99 (4.61%)	135 (7.87%)	38 (6.47%)
Chinese AD WGS cohort 2 (*N* = 1,077)			
	AD	NC	MCI
Number of participants	356	653	68
Mean age (SD)	80.31 (6.04)	78.78 (5.70)	76.97 (5.17)
Female (%)	242 (67.97%)	311 (47.62%)	36 (52.94%)
Mean MoCA score (SD)	12.65 (5.43)	23.70 (2.80)	19.04 (5.27)
Mean years of education (SD)	4.79 (4.66)	8.13 (5.03)	-
APOE-ε4 carriers (Allele frequency)	132 (20.22%)	96 (7.73%)	19 (16.18%)
APOE-ε2 carriers (Allele frequency)	36 (5.34%)	115 (9.34%)	12 (9.56%)

AD, Alzheimer's disease; ADC, National Institute on Aging Alzheimer's Disease Centers cohort; ADNI, Alzheimer's Disease Neuroimaging Initiative cohort; LOAD, Late Onset Alzheimer's Disease Family Study cohort; MCI, mild cognitive impairment; MMSE, Mini–Mental State Examination; MoCA, Montreal Cognitive Assessment; NC, normal control; SD, standard deviation; WGS, whole-genome sequencing.

Supplementary Table 2. Numbers of variants used for polygenic score analysis (for Figure 1)

1. Polygenic risk score analysis in the European-descent cohorts (Sites were selected based on Jansen et al., 2019)			
p-value groups	<1E−4	<1E−6	<1E−8
Raw			
All variants	8,100	2,959	1,799
APOE excluded	6,814	2,093	1,068
LD clumping			
All variants	1,022	314	202
APOE excluded	769	138	62
2. Polygenic risk score analysis in the European-descent cohorts (Sites were selected based on both Jansen et al., 2019 & Kunkle, 2019)			
p-value groups	<1E−4	<1E−6	<1E−8
Raw			
All variants	7,841	2,881	1,751
APOE excluded	6,608	2,055	1,054
LD clumping			
All variants	929	298	192
APOE excluded	694	134	162
3. Polygenic risk score analysis in the Chinese WGS1 and WGS2 datasets (Sites were selected based on Jansen et al., 2019)			
p-value groups	<1E−4	<1E−6	<1E−8
Raw			
All variants	7,261	2,742	1,645
APOE excluded	6,249	2037	1,047
LD-clumping			
All variants	821	288	170
APOE excluded	653	168	72
4. Polygenic risk score analysis in the Chinese WGS1 and WGS2 datasets (Sites were selected based on Jansen et al., 2019 & Zhou et al., 2018)			
p-value groups	<1E−4	<1E−6	<1E−8
Raw			
All variants	4,719	2,009	1,283
APOE excluded	4,017	1,504	837
LD clumping			
All variants	549	204	122
APOE excluded	443	128	57

LD, linkage disequilibrium; WGS, whole-genome sequencing; WGS1, Chinese WGS cohort 1; WGS2, Chinese WGS cohort 2;

Supplementary Table 3. Performance of the weighted polygenic risk score models for disease classification accuracy in the European-descent cohorts (for Figure 1)

**1. Weighted polygenic risk score model based on Jansen et al., 2019**			
p-value groups	<1E−4	<1E−6	<1E−8
Raw			
All variants	0.6363 (0.6261–0.6465)	0.6559 (0.6458–0.6659)	0.6676 (0.6576–0.6775)
APOE excluded	0.5467 (0.5360–0.5575)	0.5430 (0.5323–0.5537)	0.5410 (0.5302–0.5517)
LD clumping			
All variants	**0.6779 (0.6680–0.6877)**	0.6771 (0.6672–0.6870)	0.6767 (0.6668–0.6866)
APOE excluded	0.5795 (0.5688–0.5901)	0.5696 (0.5589–0.5802)	0.5717 (0.5611–0.5823)
**2. Weighted polygenic risk score model based on Kunkle et al., 2019**			
p-value groups	<1E−4	<1E−6	<1E−8
Raw			
All variants	0.6324 (0.6221–0.6426)	0.6423 (0.6321–0.6525)	0.6531 (0.6430–0.6632)
APOE excluded	0.5510 (0.5403–0.5617)	0.5430 (0.5323–0.5538)	0.5386 (0.5278–0.5493)
LD clumping			
All variants	**0.6785 (0.6686–0.6883)**	0.6715 (0.6616–0.6815)	0.6710 (0.6610–0.6809)
APOE excluded	0.6000 (0.5895–0.6105)	0.5762 (0.5656–0.5868)	0.5723 (0.5617–0.5830)

Classification accuracy was measured as auROC. auROC, area under the receiver operating characteristic curve; LD, linkage disequilibrium.

Supplementary Table 4. Performance of the modified weighted polygenic risk score models for disease classification accuracy in the European-descent cohorts (for Supplementary Figure 1)

**1. LDpred** (1,149 out of 8,100 sites were filtered by the software)			
p-value groups	<1E−4	<1E−6	<1E−8
Raw			
All variants	0.6208 (0.6105–0.6311)	N/A	0.6436 (0.6334–0.6538)
APOE excluded	0.5510 (0.5403–0.5617)	N/A	0.5423 (0.5316–0.5530)
**2. Winner's curse correction**			
Raw			
All variants	0.6779 (0.6680–0.6878)	**0.6796 (0.6697–0.6894)**	0.6794 (0.6696–0.6893)
APOE excluded	0.5467 (0.5360–0.5575)	0.5447 (0.5340–0.5554)	0.5481 (0.5374–0.5589)
LD clumping			
All variants	0.6779 (0.6680–0.6877)	0.6779 (0.6680–0.6877)	0.6773 (0.6675–0.6872)
APOE excluded	0.5795 (0.5688–0.5901)	0.5696 (0.5589–0.5802)	0.5717 (0.5611–0.5823)
**3. AnnoPred** (6,860 out of 8,100 sites were filtered by the software, no output)			
**4. SBayesR**			
p-value groups	<1E−4	<1E−6	<1E−8
Raw			
All variants	0.6699 (0.6597–0.6800)	0.6659 (0.6557–0.6760)	0.6663 (0.6561–0.6765)
APOE excluded	0.5876 (0.5770–0.5981)	0.5706 (0.5599–0.5812)	0.5649 (0.5543–0.5756)

Classification accuracy was measured as auROC. auROC, area under the receiver operating characteristic curve; LD, linkage disequilibrium; N/A, not applicable.

Supplementary Table 5. Evaluation of different prediction models for disease classification accuracy in the European-descent cohorts without validation (for Supplementary Figure 2)

**1. Weighted polygenic risk score model**			
p-value groups	<1E−4	<1E−6	<1E−8
Raw			
All variants	0.6720 (0.6621–0.6819)	0.6699 (0.6600–0.6798)	0.6761 (0.6662–0.6859)
APOE excluded	0.5767 (0.5661–0.5873)	0.5533 (0.5426–0.5640)	0.5464 (0.5357–0.5571)
LD clumping			
All variants	0.7086 (0.6991–0.7181)	0.6919 (0.6822–0.7016)	0.6890 (0.6792–0.6987)
APOE excluded	0.6437 (0.6336–0.6539)	0.5896 (0.5790–0.6001)	0.5750 (0.5644–0.5856)
**2. Lasso regression model**			
p-value groups	<1E−4	<1E−6	<1E−8
Raw			
All variants	0.9353 (0.9309–0.9397)	0.8313 (0.8238–0.8388)	0.7909 (0.7826–0.7991)
APOE excluded	0.8905 (0.8846–0.8963)	0.7267 (0.7174–0.7360)	0.6668 (0.6568–0.6767)
LD clumping			
All variants	0.7894 (0.7811–0.7977)	0.7407 (0.7316–0.7498)	0.7285 (0.7193–0.7378)
APOE excluded	0.6996 (0.6899–0.7092)	0.6154 (0.6050–0.6258)	0.5929 (0.5824–0.6035)
**3. Neural network model**			
p-value groups	<1E−4	<1E−6	<1E−8
Raw			
All variants	**1 (1.0000–1.0000)**	1 (1.0000–1.0000)	0.9981 (0.9970–0.9992)
APOE excluded	0.9997 (0.9992–1.0000)	1 (1.0000–1.0000)	0.9051 (0.8992–0.9110)
LD clumping			
All variants	0.9926 (0.9904–0.9949)	1 (1.0000–1.0000)	1 (1.0000–1.0000)
APOE excluded	0.8624 (0.8556–0.8692)	0.9390 (0.9329–0.9450)	0.7464 (0.7376–0.7553)

Classification accuracy was measured as auROC. auROC, area under the receiver operating characteristic curve; lasso, least absolute shrinkage and selection operator; LD, linkage disequilibrium.

Supplementary Table 6. Evaluation of different prediction models for disease classification accuracy in the European-descent cohorts using the five-fold cross-validation method (for Supplementary Figure 3)

**1. Weighted polygenic risk score model**			
p-value groups	<1E−4	<1E−6	<1E−8
Raw			
All variants	0.6548 (0.6541–0.6555)	0.6622 (0.6619–0.6626)	0.6710 (0.6708–0.6713)
APOE excluded	0.5572 (0.5565–0.5579)	0.5449 (0.5444–0.5454)	0.5411 (0.5406–0.5416)
LD clumping			
All variants	0.6904 (0.6900–0.6908)	0.6857 (0.6855–0.6859)	0.6849 (0.6848–0.6851)
APOE excluded	0.5960 (0.5949–0.5970)	0.5761 (0.5754–0.5767)	0.5674 (0.5669–0.5679)
**2. Lasso regression model**			
p-value groups	<1E−4	<1E−6	<1E−8
Raw			
All variants	0.7208 (0.7199–0.7217)	0.7160 (0.7155–0.7166)	0.7129 (0.7126–0.7132)
APOE excluded	0.5995 (0.5972–0.6019)	0.5843 (0.5831–0.5854)	0.5782 (0.5771–0.5793)
LD clumping			
All variants	0.7162 (0.7153–0.7170)	0.7130 (0.7127–0.7133)	0.7094 (0.7092–0.7095)
APOE excluded	0.5929 (0.5904–0.5954)	0.5827 (0.5814–0.5841)	0.5745 (0.5733–0.5757)
**3. Neural network model**			
p-value groups	<1E−4	<1E−6	<1E−8
Raw			
All variants	**0.7260 (0.7251–0.7270)**	0.7256 (0.7352–0.7260)	0.7253 (0.7245–0.7261)
APOE excluded	0.5799 (0.5775–0.5823)	0.5898 (0.5883–0.5913)	0.5812 (0.5800–0.5825)

Classification accuracy was measured as auROC. auROC, area under the receiver operating characteristic curve; lasso, least absolute shrinkage and selection operator; LD, linkage disequilibrium.

Supplementary Table 7. Evaluation of different prediction models for disease classification accuracy in independent European-descent cohorts (for Figures 2a–d, Supplementary Figure 4)

auROC (95% CI)			
**1. Weighted polygenic risk score model**			
p-value groups	<1E−4	<1E−6	<1E−8
Raw			
ADC	0.6414 (0.6263–0.6565)	0.6651 (0.6504–0.6799)	0.6711 (0.6566–0.6857)
LOAD	0.6330 (0.6164–0.6496)	0.6457 (0.6292–0.6622)	0.6519 (0.6355–0.6682)
ADNI (testing)	0.6293 (0.6001–0.6585)	0.6591 (0.6305–0.6877)	0.6632 (0.6346–0.6918)
LD clumping			
ADC	0.7003 (0.6860–0.7145)	0.7015 (0.6872–0.7157)	0.7005 (0.6863–0.7148)
LOAD	0.6735 (0.6575–0.6894)	0.6657 (0.6496–0.6818)	0.6652 (0.6491–0.6814)
ADNI (testing)	0.6747 (0.6464–0.7030)	0.6831 (0.6550–0.7112)	0.6835 (0.6554–0.7116)
**2. Lasso regression model**			
ADC	0.8074 (0.7953–0.8194)	0.8159 (0.8039–0.8279)	0.7894 (0.7768–0.8020)
LOAD	0.7939 (0.7807–0.8072)	0.7874 (0.7738–0.8009)	0.7615 (0.7473–0.7757)
ADNI (testing)	0.6947 (0.6671–0.7224)	0.6555 (0.6269–0.6841)	0.6793 (0.6511–0.7074)
**3. Neural network model**			
ADC	**0.8438 (0.8329–0.8547)**	0.8273 (0.8160–0.8387)	0.8156 (0.8039–0.8274)
LOAD	**0.8315 (0.8194–0.8437)**	0.8162 (0.8035–0.8290)	0.7995 (0.7862–0.8127)
ADNI (testing)	**0.6956 (0.6678–0.7233)**	0.6892 (0.6614–0.7170)	0.6853 (0.6574–0.7131)
auPRC			
**1. Weighted polygenic risk score model**			
p-value groups	<1E−4	<1E−6	<1E−8
Raw			
ADC	0.7991	0.8149	0.8205
LOAD	0.6150	0.6221	0.6247
ADNI (testing)	0.6226	0.6569	0.6638
LD clumping			
ADC	0.8377	0.8371	0.8362
LOAD	0.6413	0.6291	0.6291
ADNI (testing)	0.6637	0.6708	0.6690
**2. Lasso regression model**			
ADC	0.8956	0.8929	0.8820
LOAD	0.7696	0.7565	0.7376
ADNI (testing)	0.7014	0.6340	0.6577
**3. Neural network model**			
ADC	**0.9155**	0.9095	0.9030
LOAD	**0.8147**	0.8054	0.7909
ADNI (testing)	**0.7016**	0.6832	0.6909

Classification accuracy was measured as auROC or auPRC. AD, Alzheimer's disease; ADC, National Institute on Aging Alzheimer's Disease Centers cohort; ADNI, Alzheimer's Disease Neuroimaging Initiative cohort; auPRC, area under the precision-recall curve; auROC, area under the receiver operating characteristic curve; CI, confidence interval; lasso, least absolute

shrinkage and selection operator; LD, linkage disequilibrium; LOAD, Late Onset Alzheimer's Disease Family Study cohort.

Supplementary Table 8. Evaluation of different prediction models for disease classification accuracy in independent European-descent cohorts removing potential duplicate samples (for Supplementary Figure 5)

auROC (95% CI)			
**1. Weighted polygenic risk score model**			
p-value groups	<1E−4	<1E−6	<1E−8
wPRS			
ADC	0.6999 (0.6857–0.7142)	0.7011 (0.6869–0.7154)	0.7001 (0.6859–0.7144)
LOAD	0.6642 (0.6474–0.6810)	0.6570 (0.6401–0.6739)	0.6560 (0.6391–0.6730)
ADNI (testing)	0.6672 (0.6382–0.6963)	0.6770 (0.6482–0.7058)	0.6770 (0.6482–0.7058)
wPRS2			
ADC	0.7021 (0.6878–0.7165)	0.6950 (0.6806–0.7095)	0.6939 (0.6794–0.7084)
LOAD	0.6614 (0.6446–0.6782)	0.6520 (0.6350–0.6690)	0.6512 (0.6342–0.6682)
ADNI (testing)	0.6736 (0.6448–0.7024)	0.6711 (0.6422–0.7000)	0.6695 (0.6406–0.6984)
**2. Lasso regression model**			
ADC	0.8072 (0.7951–0.8192)	0.8157 (0.8036–0.8277)	0.7892 (0.7765–0.8018)
LOAD	0.7814 (0.7672–0.7956)	0.7764 (0.7620–0.7909)	0.7496 (0.7345–0.7646)
ADNI (testing)	0.6893 (0.6610–0.7176)	0.6515 (0.6222–0.6808)	0.6790 (0.6503–0.7077)
**3. Neural network model**			
ADC	0.8437 (0.8328–0.8546)	0.8270 (0.8156–0.8383)	0.8152 (0.8035–0.8269)
LOAD	0.8202 (0.8071–0.8333)	0.8055 (0.7918–0.8191)	0.7888 (0.7747–0.8029)
ADNI (testing)	0.6891 (0.6606–0.7175)	0.6827 (0.6542–0.7112)	0.6801 (0.6516–0.7087)
auPRC			
**1. Weighted polygenic risk score model**			
p-value groups	<1E−4	<1E−6	<1E−8
wPRS			
ADC	0.8373	0.8366	0.8358
LOAD	0.6478	0.6366	0.6367
ADNI (testing)	0.6548	0.6621	0.6594
wPRS2			
ADC	0.8357	0.8306	0.8302
LOAD	0.6454	0.6328	0.6325
ADNI (testing)	0.6579	0.6566	0.6524
**2. Lasso regression model**			
ADC	0.8954	0.8927	0.8818
LOAD	0.7692	0.7593	0.7397
ADNI (testing)	0.6922	0.6275	0.6536
**3. Neural network model**			
ADC	0.9153	0.9092	0.9026
LOAD	0.8134	0.8035	0.7902
ADNI (testing)	0.6921	0.6724	0.6823

Classification accuracy was measured as auROC or auPRC. AD, Alzheimer's disease; ADC, National Institute on Aging Alzheimer's Disease Centers cohort; ADNI, Alzheimer's Disease Neuroimaging Initiative cohort; auPRC, area under the precision-recall curve; auROC, area under the receiver operating characteristic curve; CI, confidence interval; lasso, least absolute shrinkage and selection operator; LOAD, Late Onset Alzheimer's Disease Family Study cohort; wPRS, weighted polygenic risk score.

Supplementary Table 9. Evaluation of different prediction models for disease classification accuracy in the European-descent cohorts with respect to different ancestral origins (for Supplementary Figure 6)

$p < 1E{-}4$	*n*	wPRS (all sites after clumping)	Lasso	Neural network
auROC				
**European**	9,940	**0.6889 (0.6784–0.6993)**	0.7754 (0.7663–0.7846)	0.8105 (0.8020–0.8190)
**African-American**	713	0.5960 (0.5544–0.6376)	**0.8134 (0.7822–0.8447)**	**0.8389 (0.8105–0.8673)**
**Latin-American**	604	0.5979 (0.5514–0.6445)	0.7526 (0.7104–0.7948)	0.7672 (0.7269–0.8075)
auPRC				
**European**	9,940	0.7484	0.8225	0.8505
**African-American**	713	0.6260	0.8296	0.8630
**Latin-American**	604	0.7500	0.8293	0.8492

auPRC, area under the precision-recall curve; auROC, area under the receiver operating characteristic curve; lasso, least absolute shrinkage and selection operator; *n*, number of sites; wPRS, weighted polygenic risk score.

Supplementary Table 10. Evaluation of different prediction models for disease classification accuracy in the European-descent population stratified by sex (for Supplementary Figure 7)

$p < 1E{-}4$	n	wPRS (all sites after clumping)	Lasso	Neural network
auROC				
**Male**	4,230	0.6888 (0.6727–0.7049)	0.7789 (0.7648–0.7929)	0.8048 (0.7915–0.8181)
**Female**	5,710	0.6881 (0.6744–0.7018)	0.7727 (0.7605–0.7848)	0.8139 (0.8028–0.8249)
auPRC				
**Male**	4,230	0.7712	0.8428	0.8605
**Female**	5,710	0.7295	0.8061	0.8424

auPRC, area under the precision-recall curve; auROC, area under the receiver operating characteristic curve; lasso, least absolute shrinkage and selection operator; *n*, number of sites; wPRS, weighted polygenic risk score.

Supplementary Table 11. Evaluation of different prediction models for disease classification accuracy in the European-descent population by age group (for Supplementary Figure 8)

p < 1E−4	*n*	wPRS (all sites after clumping)	Lasso	Neural network
auROC				
**AGE < 72**	2,084	0.6505 (0.6268–0.6743)	0.7315 (0.7098–0.7532)	0.7736 (0.7533–0.7940)
**72 ≤ AGE < 78**	1,830	0.7285 (0.7056–0.7514)	0.8089 (0.7893–0.8285)	0.8397 (0.8217–0.8577)
**78 ≤ AGE < 83**	1,969	0.7163 (0.6929–0.7397)	0.7994 (0.7794–0.8195)	0.8388 (0.8209–0.8568)
**83 ≤ AGE < 89**	2,154	0.6957 (0.6726–0.7188)	0.7930 (0.7733–0.8126)	0.8248 (0.8066–0.8430)
**89 ≤ AGE**	1,903	0.6936 (0.6689–0.7183)	0.7966 (0.7753–0.8178)	0.8119 (0.7920–0.8318)
auPRC				
**AGE < 72**	2,084	0.5765	0.6635	0.7145
**72 ≤ AGE < 78**	1,830	0.7352	0.8205	0.8398
**78 ≤ AGE < 83**	1,969	0.8216	0.8822	0.9056
**83 ≤ AGE < 89**	2,154	0.8040	0.8749	0.8925
**89 ≤ AGE**	1,903	0.8158	0.8780	0.8968

auPRC, area under the precision-recall curve; auROC, area under the receiver operating characteristic curve; lasso, least absolute shrinkage and selection operator; *n*, number of sites; wPRS, weighted polygenic risk score.

Supplementary Table 12. Evaluation of trans-ethnic effects on different prediction models for disease classification accuracy in Chinese WGS cohort 1 measured by the area under the receiver operating characteristic curve (for Supplementary Figures 9, 10)

**1. Weighted polygenic risk score model**			
p-value groups	<1E−4	<1E−6	<1E−8
a. Effect sizes from Jansen et al., 2019			
Raw			
All variants	0.5059 (0.4807–0.5311)	0.5090 (0.4837–0.5342)	0.5198 (0.4945–0.5450)
APOE excluded	0.5034 (0.4782–0.5286)	0.4953 (0.4700–0.5205)	0.5010 (0.4758–0.5262)
LD clumping			
All variants	0.5042 (0.4789–0.5294)	0.5069 (0.4816–0.5322)	0.5051 (0.4799–0.5304)
APOE excluded	0.5042 (0.4790–0.5295)	0.4933 (0.4680–0.5185)	0.5189 (0.4937–0.5441)
b. Effect sizes from Zhou et al., 2018			
Raw			
All variants	0.5727 (0.5478–0.5976)	0.6379 (0.6139–0.6620)	0.6485 (0.6247–0.6723)
APOE excluded	0.5255 (0.5003–0.5508)	0.5566 (0.5315–0.5816)	0.5504 (0.5252–0.5755)
LD clumping			
All variants	0.6628 (0.6395–0.6862)	0.6566 (0.6330–0.6801)	0.6615 (0.6381–0.6850)
APOE excluded	0.5819 (0.5571–0.6067)	0.5539 (0.5288–0.5789)	0.5475 (0.5225–0.5726)
c. Effect sizes from WGS1			
Raw			
All variants	0.6854 (0.6626–0.7083)	0.6656 (0.6423–0.6890)	0.6656 (0.6423–0.6890)
APOE excluded	0.6155 (0.5911–0.6398)	0.5767 (0.5517–0.6016)	0.5658 (0.5408–0.5908)
LD clumping			
All variants	0.7552 (0.7343–0.7760)	0.6958 (0.6731–0.7184)	0.6863 (0.6634–0.7092)
APOE excluded	0.7099 (0.6877–0.7321)	0.6154 (0.5911–0.6397)	0.6044 (0.5798–0.6289)
**2. Lasso regression model**			
p-value groups	<1E−4	<1E−6	<1E−8
a. Model from European-descent cohorts			
All variants	0.6701 (0.6468–0.6933)	0.6799 (0.6569–0.7029)	0.6763 (0.6532–0.6994)
APOE excluded	0.5589 (0.5338–0.5840)	0.5712 (0.5462–0.5961)	0.5718 (0.5470–0.5967)
b. Model from WGS1			
All variants	0.7185 (0.6965–0.7406)	0.7016 (0.6791–0.7241)	0.6921 (0.6694–0.7149)
APOE excluded	0.6005 (0.5758–0.6251)	0.5900 (0.5653–0.6147)	0.5872 (0.5624–0.6119)
**3. Neural network model**			
p-value groups	<1E−4	<1E−6	<1E−8
a. Model from European-descent cohorts			
All variants	0.6533 (0.6297–0.6769)	0.6655 (0.6421–0.6888)	0.6676 (0.6443–0.6909)
APOE excluded	0.5397 (0.5146–0.5649)	0.5583 (0.5332–0.5834)	0.5456 (0.5205–0.5708)
b. Model from WGS1			
All variants	0.7492 (0.7280–0.7703)	0.7465 (0.7252–0.7678)	0.7337 (0.7121–0.7553)
APOE excluded	0.6838 (0.6608–0.7067)	0.5781 (0.5533–0.6030)	0.5667 (0.5417–0.5917)

Lasso, least absolute shrinkage and selection operator; LD, linkage disequilibrium; WGS, whole-genome sequencing; WGS1, Chinese WGS cohort 1.

Supplementary Table 13. Evaluation of the trans-ethnic effects on different prediction models for disease classification accuracy in Chinese WGS cohort 1 measured by the area under the precision-recall curve (for Supplementary Figures 9, 10)

**1. Weighted polygenic risk score model**			
p-value groups	<1E−4	<1E−6	<1E−8
a. Effect sizes from Jansen et al., 2019			
Raw			
All variants	0.5522	0.5458	0.5374
APOE excluded	0.5589	0.5539	0.5484
LD clumping			
All variants	0.5447	0.5454	0.5478
APOE excluded	0.5499	0.5501	0.5712
b. Effect sizes from Zhou et al., 2018			
Raw			
All variants	0.6108	0.6714	0.6894
APOE excluded	0.5716	0.5964	0.5908
LD clumping			
All variants	0.7086	0.7018	0.7062
APOE excluded	0.6230	0.5938	0.5945
c. Effect sizes from the WGS1 dataset			
Raw			
All variants	0.7270	0.7110	0.7137
APOE excluded	0.6589	0.6145	0.6079
LD clumping			
All variants	0.7800	0.7325	0.7239
APOE excluded	0.7527	0.6571	0.6445
**2. Lasso regression model**			
p-value groups	<1E−4	<1E−6	<1E−8
a. Model from European-descent cohorts			
All variants	0.7160	0.7248	0.7255
APOE excluded	0.5864	0.6069	0.6142
b. Model from WGS1			
All variants	0.7499	0.7359	0.7313
APOE excluded	0.6332	0.6268	0.6234
**3. Neural network model**			
p-value groups	<1E−4	<1E−6	<1E−8
a. Model from European-descent cohorts			
All variants	0.7067	0.7156	0.7175
APOE excluded	0.5870	0.5921	0.5893
b. Model from WGS1			
All variants	0.7822	0.7765	0.7606
APOE excluded	0.7304	0.6177	0.6074

LD, linkage disequilibrium; WGS, whole-genome sequencing; WGS1, Chinese WGS cohort 1.

Supplementary Table 14. Evaluation of the trans-ethnic effects on different prediction models for disease classification accuracy in Chinese WGS cohort 2 measured by the area under the receiver operating characteristic curve (for Supplementary Figures 9, 10)

**1. Weighted polygenic risk score model**			
p-value groups	<1E−4	<1E−6	<1E−8
a. Effect sizes from Jansen et al., 2019			
Raw			
All variants	0.5185 (0.4809–0.5560)	0.4888 (0.4514–0.5262)	0.5136 (0.4761–0.5510)
APOE excluded	0.5211 (0.4837–0.5585)	0.5130 (0.4756–0.5504)	0.4918 (0.4543–0.5293)
LD clumping			
All variants	0.5046 (0.4667–0.5424)	0.4992 (0.4613–0.5370)	0.5043 (0.4663–0.5422)
APOE excluded	0.5394 (0.5018–0.5770)	0.5336 (0.4967–0.5706)	0.5161 (0.4787–0.5535)
b. Effect sizes from Zhou et al., 2018			
Raw			
All variants	0.5460 (0.5082–0.5838)	0.5762 (0.5385–0.6140)	0.5945 (0.5571–0.6320)
APOE excluded	0.5183 (0.4802–0.5563)	0.5328 (0.4953–0.5704)	0.5437 (0.5064–0.5811)
LD clumping			
All variants	0.5979 (0.5603–0.6355)	0.5919 (0.5543–0.6295)	0.6030 (0.5652–0.6407)
APOE excluded	0.5564 (0.5187–0.5941)	0.5311 (0.4938–0.5685)	0.5274 (0.4898–0.5651)
c. Effect sizes from the WGS1 dataset			
Raw			
All variants	0.5850 (0.5482–0.6219)	0.5948 (0.5578–0.6319)	0.6071 (0.5703–0.6438)
APOE excluded	0.5365 (0.4996–0.5735)	0.5266 (0.4894–0.5638)	0.5289 (0.4915–0.5662)
LD clumping			
All variants	0.6207 (0.5842–0.6572)	0.6227 (0.5861–0.6592)	0.6273 (0.5907–0.6639)
APOE excluded	0.5496 (0.5130–0.5862)	0.5363 (0.4993–0.5733)	0.5315 (0.4943–0.5688)
**2. Lasso regression model**			
p-value groups	<1E−4	<1E−6	<1E−8
a. Model from European-descent cohorts			
All variants	0.6481 (0.6119–0.6843)	0.6586 (0.6228–0.6944)	0.6439 (0.6075–0.6803)
APOE excluded	0.5612 (0.5240–0.5984)	0.5791 (0.5422–0.6160)	0.5680 (0.5307–0.6053)
b. Model from the WGS1 dataset			
All variants	0.6290 (0.5923–0.6657)	0.6182 (0.5807–0.6557)	0.6225 (0.5853–0.6596)
APOE excluded	0.5052 (0.4678–0.5427)	0.4948 (0.4577–0.5320)	0.5123 (0.4749–0.5498)
**3. Neural network model**			
p-value groups	<1E−4	<1E−6	<1E−8
a. Model from European-descent cohorts			
All variants	0.6329 (0.5962–0.6697)	0.6441 (0.6075–0.6807)	0.6312 (0.5940–0.6684)
APOE excluded	0.5539 (0.5168–0.5911)	0.5648 (0.5279–0.6018)	0.5282 (0.4908–0.5655)
b. Model from the WGS1 dataset			
All variants	0.6093 (0.5720–0.6465)	0.6069 (0.5693–0.6446)	0.6151 (0.5778–0.6524)
APOE excluded	0.5332 (0.4959–0.5704)	0.5281 (0.4906–0.5655)	0.5297 (0.4922–0.5672)

LD, linkage disequilibrium; WGS, whole-genome sequencing; WGS1, Chinese WGS cohort 1; WGS2, Chinese WGS cohort 2.

Supplementary Table 15. Evaluation of the trans-ethnic effects on different prediction models for disease classification in Chinese WGS cohort 2 measured by the area under the precision-recall curve (for Supplementary Figures 9, 10)

**1. Weighted polygenic risk score model**			
p-value groups	<1E−4	<1E−6	<1E−8
1. Effect sizes from Jansen et al., 2019			
Raw			
All variants	0.3459	0.3549	0.3517
APOE excluded	0.3415	0.3529	0.3504
LD clumping			
All variants	0.3560	0.3639	0.3662
APOE excluded	0.3329	0.3267	0.3379
2. Effect sizes from Zhou et al., 2018			
Raw			
All variants	0.4035	0.4426	0.4622
APOE excluded	0.3829	0.3763	0.3814
LD clumping			
All variants	0.4727	0.4672	0.4789
APOE excluded	0.4019	0.3820	0.3826
3. Effect sizes from the WGS1 dataset			
Raw			
All variants	0.4321	0.4495	0.4636
APOE excluded	0.3730	0.3699	0.3754
LD clumping			
All variants	0.4937	0.4930	0.4952
APOE excluded	0.3806	0.3738	0.3763
**2. Lasso regression model**			
p-value groups	<1E−4	<1E−6	<1E−8
1. Model from European-descent cohorts			
All variants	0.5245	0.5245	0.5216
APOE excluded	0.4000	0.4179	0.4089
2. Model from the WGS1 dataset			
All variants	0.4986	0.5009	0.5008
APOE excluded	0.3493	0.3575	0.3715
**3. Neural network model**			
p-value groups	<1E−4	<1E−6	<1E−8
1. Model from European-descent cohorts			
All variants	0.5030	0.5073	0.5038
APOE excluded	0.3974	0.3966	0.3761
2. Model from the WGS1 dataset			
All variants	0.4688	0.4788	0.4905
APOE excluded	0.3692	0.3751	0.3802

LD, linkage disequilibrium; WGS, whole-genome sequencing; WGS1, Chinese WGS cohort 1; WGS2, Chinese WGS cohort 2.

Supplementary Table 16. Sources of the variants for the replication analysis

Ethnicity	Index	Sample size	PMID	First author	DOI	Year
European-descent	Number of variants retrieved: 183					
	1	788,989	N/A	Céline Bellenguez	10.1101/2020.10.01.20200659	2020[8]
	2	472,868	N/A	Jeremy Schwartzentruber	10.1101/2020.01.22.20018424	2020[9]
	3	1,126,563	N/A	Douglas P. Wightman	10.1101/2020.11.20.20235275	2020[10]
	4	455,258	30617256	Iris E. Jansen	10.1038/s41588-018-0311-9	2019[11]
	5	94,437	30820047	Brian W. Kunkle	10.1038/s41588-019-0358-2	2019[12]
	6	25,580	29777097	Riccardo E. Marioni	10.1038/s41398-018-0150-6	2018[13]
African-American	Number of variants retrieved: 14					
	7	8,006	33074286	Brian W. Kunkle	10.1001/jamaneurol.2020.3536	2020[14]
Asian	Number of variants retrieved: 13					
	8	11,506	33188687	Longfei Jia	10.1093/brain/awaa364	2020[15]
Mixed	Number of variants retrieved: 6					
	9	59,556	28183528	Gyungah R. Jun	10.1016/j.jalz.2016.12.012	2017[16]

DOI, digital object identifier; PMID, PubMed unique identifier; N/A, not applicable.

Supplementary Table 17. Performance of the polygenic score models for disease classification accuracy in the Chinese whole-genome sequencing cohorts (for Figure 3a, Supplementary Figure 11)

Models	Chinese AD WGS cohort 1		Chinese AD WGS cohort 2
	**AD vs. NC** (1,116 AD, 915 NC)	**MCI vs. NC** (309 MCI, 915 NC)	**AD vs. NC** (356 AD, 653 NC)
auROC (95% confidence interval)			
**Weighted polygenic risk score model**			
wPRS	0.6686 (0.6453–0.6918)	0.5688 (0.5295–0.6080)	0.6219 (0.5851–0.6587)
**Lasso regression model**			
Lasso	0.7069 (0.6840–0.7298)	0.5963 (0.5574–0.6327)	**0.6348** (0.5997–0.6700)
Lasso_APOE	0.6528 (0.6297–0.6761)	0.5777 (0.5406–0.6158)	0.6323 (0.5959–0.6697)
Lasso_nonAPOE	0.6114 (0.5874–0.6354)	0.5412 (0.5050–0.5786)	0.5539 (0.5187–0.5904)
**Neural network model**			
NN	**0.7718** (0.7507–0.7923)	**0.6241** (0.5867–0.6614)	0.6299 (0.5924–0.6656)
auPRC			
**Weighted polygenic risk score model**			
wPRS	0.7120	0.3399	0.4894
**Lasso regression model**			
Lasso	0.7391	0.3497	0.5112
Lasso_APOE	0.7059	0.3331	0.4977
Lasso_nonAPOE	0.6533	0.2743	0.3911
**Neural network model**			
NN	**0.7706**	**0.3620**	**0.5258**

Classification accuracy was measured as auROC or auPRC. AD, Alzheimer's disease; auPRC, precision-recall curve; area under the auROC, area under the receiver operating characteristic curve; lasso, least absolute shrinkage and selection operator; Lasso_APOE, lasso model constructed using variants in *APOE* regions; lasso_nonAPOE, lasso model constructed using variants outside of *APOE* regions; MCI, mild cognitive impairment; NC, normal control; NN, neural network; WGS, whole-genome sequencing; wPRS, weighted polygenic risk score.

Supplementary Table 18. Evaluation of different prediction models using 37 variants for disease classification accuracy using the five-fold cross-validation method (for Supplementary Figure 13)

Model	European-descent population (*n* = 11,352)	
	auROC (95% CI)	auPRC (95% CI)
wPRS	0.6839 (0.6837–0.6840)	0.7579 (0.7578–0.7580)
Lasso	0.7075 (0.7072–0.7079)	0.7709 (0.7706–0.7712)
NN	**0.7235 (0.7230–0.7241)**	**0.7944 (0.7938–0.7950)**
	Chinese population (WGS1; *n* = 2,031)	
wPRS	0.6632 (0.6622–0.6642)	0.7087 (0.7082–0.7092)
Lasso	0.6844 (0.6820–0.6868)	0.7211 (0.7196–0.7225)
NN	**0.6872 (0.6856–0.6889)**	**0.7293 (0.7276–0.7309)**

auPRC, area under the precision-recall curve; area under the auROC, area under the receiver operating characteristic curve; CI, confidence interval; lasso, least absolute shrinkage and selection operator; *n*, number of samples; NN, neural network; WGS, whole-genome sequencing; WGS1, Chinese WGS cohort 1; wPRS, weighted polygenic risk score;

Supplementary Table 19. Evaluation of the trans-ethnic effects on different prediction models using 37 variants for disease classification (for Supplementary Figure 16)

	Models from European-descent population data			
Model	European-descent datasets (*n* =11,352)		WGS1 dataset (*n* = 2,031)	
	auROC (95% CI)	auPRC	auROC (95% CI)	auPRC
wPRS (Jansen et al.)	0.6840 (0.6743–0.6938)	0.7578	0.6603 (0.6368–0.6838)	0.7082
Lasso	0.7115 (0.7020–0.7210)	0.7745	0.6730 (0.6498–0.6962)	0.7195
NN	**0.7502 (0.7412–0.7592)**	**0.8041**	0.6021 (0.5917–0.6125)	0.6938
	Models from Chinese data			
	European-descent datasets (*n* =11,352)		WGS1 dataset (*n* = 2,031)	
wPRS (WGS1)	0.6537 (0.6436–0.6637)	0.7346	0.6686 (0.6453–0.6918)	0.7120
Lasso	0.6181 (0.6079–0.6284)	0.7150	0.7069 (0.6845–0.7294)	0.7391
NN	0.6496 (0.6259–0.6733)	0.7000	**0.7718 (0.7510–0.7925)**	**0.7706**

auPRC, area under the precision-recall curve; area under the auROC, area under the receiver operating characteristic curve; CI, confidence interval; lasso, least absolute shrinkage and selection operator; *n*, number of samples; NN, neural network; WGS, whole-genome sequencing; WGS1, Chinese WGS cohort 1; wPRS, weighted polygenic risk score.

Supplementary Table 20. Stratification of individual disease risk based on polygenic scores and their associations with phenotypes (for Figure 3d, Supplementary Figure 18)

	**Chinese AD WGS cohort 1**			**Chinese AD WGS cohort 2**	
	**AD**	**MCI**	**NC**	**AD**	**NC**
**Low risk (*n*)**	155	101	443	90	261
**Medium risk (*n*)**	466	120	357	148	287
**High risk (*n*)**	495	88	115	118	105
**Dataset**	**Phenotype**	**Group**	**β**	**SE**	***p*-value**
**Chinese AD WGS cohort 1**	AD vs. NC	High vs. Low	2.525	0.141	**<2E−16**
		High vs. Medium	1.191	0.126	**<2E−16**
		Medium vs. Low	1.320	0.117	**<2E−16**
	MCI vs. NC	High vs. Low	1.263	0.184	**6.16E−12**
		High vs. Medium	0.819	0.179	**5.06E−06**
		Medium vs. Low	0.384	0.153	**1.25E−02**
**Chinese AD WGS cohort 2**	AD vs. NC	High vs. Low	1.322	0.272	**1.13E−06**
		High vs. Medium	0.982	0.258	**1.44E−04**
		Medium vs. Low	0.384	0.238	0.107

AD, Alzheimer's disease; β, effect size; MCI, mild cognitive impairment; NC, normal control; SE, standard error; WGS, whole-genome sequencing. Significant associations ($p < 0.05$) are displayed in bold text.

Supplementary Table 21. Association between polygenic scores and cognitive performance (for Figures 3e–h, Supplementary Figure 19)

Dataset	Group	β	SE	p-value
**Chinese AD WGS cohort 1**	All participants	−1.768	0.082	**<2E−16**
	Non-AD participants	−0.298	0.082	**3.10E−04**
	APOE-ε3 carriers	−2.145	0.128	**<2E−16**
	APOE-ε4 carriers	−1.753	0.335	**2.18E−07**
**Chinese AD WGS cohort 2**	All participants	−0.287	0.107	**7.38E−03**
	Non-AD participants	0.171	0.135	0.206
	APOE-ε3 carriers	−0.071	0.166	0.668
	APOE-ε4 carriers	0.116	0.452	0.798

AD, Alzheimer's disease; β, effect size; SE, standard error; WGS, whole-genome sequencing. Significant associations ($p < 0.05$) are displayed in bold text.

Supplementary Table 22. Associations between polygenic scores and the plasma ATN biomarker panel (for Figures 4a–d)

Group	Biomarker	NN				PRS_Full			
All		*β*	SE	*t*	*p*-value	*β*	SE	*t*	*p*-value
	$A\beta_{42}$	−3.614	0.957	−3.776	**1.96E−04**	−1.261	0.334	−3.780	**1.93E−04**
	$A\beta_{40}$	21.945	16.767	1.309	1.92E−01	7.139	6.030	1.184	2.37E−01
	$A\beta_{42}/A\beta_{40}$	−0.016	0.004	−3.628	**3.42E−04**	−0.005	0.002	−3.250	**1.30E−03**
	tau	−0.051	0.276	−0.185	8.53E−01	0.076	0.097	0.777	4.38E−01
	p-tau181	1.536	0.355	4.322	**2.20E−05**	0.618	0.141	4.379	**1.72E−05**
	NfL	5.388	2.167	2.487	**1.35E−02**	1.587	0.739	2.148	**3.26E−02**
		PRS_APOE				PRS_nonAPOE			
		β	SE	*t*	*p*-value	*β*	SE	*t*	*p*-value
	$A\beta_{42}$	−1.664	0.354	−4.708	**4.01E−06**	0.030	0.588	0.051	9.59E−01
	$A\beta_{40}$	5.355	7.355	0.728	4.67E−01	11.423	9.584	1.192	2.34E−01
	$A\beta_{42}/A\beta_{40}$	−0.007	0.002	−3.897	**1.23E−04**	0.000	0.003	0.012	9.91E−01
	tau	0.022	0.106	0.211	8.33E−01	0.203	0.150	1.352	1.78E−01
	p-tau181	0.657	0.168	3.901	**1.22E−04**	0.465	0.253	1.839	6.71E−02
	NfL	1.704	0.914	1.864	6.34E−02	0.854	1.168	0.731	4.65E−01
Group	Biomarker	NN				PRS_Full			
NC		*β*	SE	*t*	*p*-value	*β*	SE	*t*	*p*-value
	$A\beta_{42}$	−3.936	1.276	−3.086	**2.55E−03**	−1.524	0.526	−2.894	**4.55E−03**
	$A\beta_{40}$	24.432	22.467	1.087	2.79E−01	10.729	8.618	1.245	2.16E−01
	$A\beta_{42}/A\beta_{40}$	−0.023	0.007	−3.145	**2.12E−03**	−0.009	0.003	−3.022	**3.09E−03**
	tau	−0.454	0.384	−1.183	2.39E−01	−0.113	0.179	−0.633	5.28E−01
	p-tau181	0.629	0.278	2.265	**2.55E−02**	0.309	0.190	1.625	1.07E−01
	NfL	4.749	2.229	2.130	**3.53E−02**	1.110	0.811	1.368	1.74E−01
		PRS_APOE				PRS_nonAPOE			
		β	SE	*t*	*p*-value	*β*	SE	*t*	*p*-value
	$A\beta_{42}$	−2.229	0.493	−4.520	**1.52E−05**	0.336	0.879	0.383	7.03E−01
	$A\beta_{40}$	2.541	8.667	0.293	7.70E−01	30.349	12.515	2.425	**1.69E−02**
	$A\beta_{42}/A\beta_{40}$	−0.011	0.003	−3.533	**5.94E−04**	−0.004	0.006	−0.782	4.36E−01
	tau	−0.160	0.177	−0.901	3.70E−01	0.105	0.245	0.428	6.69E−01
	p-tau181	0.293	0.222	1.324	1.88E−01	0.275	0.228	1.205	2.31E−01
	NfL	0.697	0.850	0.820	4.14E−01	1.724	1.223	1.409	1.61E−01
Group	Biomarker	NN				PRS_Full			
AD		*β*	SE	*t*	*p*-value	*β*	SE	*t*	*p*-value
	$A\beta_{42}$	−2.457	1.475	−1.665	9.80E−02	−0.910	0.486	−1.872	6.32E−02
	$A\beta_{40}$	9.025	22.723	0.397	6.92E−01	−1.704	8.015	−0.213	8.32E−01
	$A\beta_{42}/A\beta_{40}$	−0.006	0.004	−1.460	1.46E−01	−0.002	0.002	−1.408	1.61E−01
	tau	0.306	0.386	0.791	4.30E−01	0.162	0.121	1.331	1.85E−01
	p-tau181	0.711	0.708	1.005	3.17E−01	0.132	0.227	0.579	5.63E−01
	NfL	0.953	3.698	0.258	7.97E−01	0.002	1.044	0.002	9.99E−01
		PRS_APOE				PRS_nonAPOE			
		β	SE	*t*	*p*-value	*β*	SE	*t*	*p*-value
	$A\beta_{42}$	−1.181	0.519	−2.276	**2.43E−02**	0.031	0.774	0.040	9.68E−01
	$A\beta_{40}$	3.290	9.693	0.339	7.35E−01	−13.761	15.093	−0.912	3.63E−01
	$A\beta_{42}/A\beta_{40}$	−0.003	0.002	−1.718	8.79E−02	0.002	0.002	0.702	4.84E−01
	tau	0.089	0.151	0.590	5.56E−01	0.274	0.191	1.432	1.54E−01
	p-tau181	0.190	0.268	0.710	4.79E−01	0.000	0.506	0.000	1.00E+00
	NfL	0.741	1.284	0.577	5.65E−01	−2.297	1.821	−1.261	2.09E−01

Aβ, amyloid-beta; AD, Alzheimer's disease; ATN, Aβ, tau, and NfL; *β*, effect size; lasso, least absolute shrinkage and selection operator; NC, normal control; NfL, neurofilament light polypeptide; NN, neural network; p-tau181, tau phosphorylated at threonine-181; PRS, polygenic risk score; PRS_APOE, lasso model constructed using variants in *APOE* regions;

PRS_nonAPOE, lasso model constructed using variants outside of *APOE* regions; SE, standard error. Bold underlined text indicates $p < 0.05$; underlined text indicates $p < 0.10$.

Supplementary Table 23. Summary of protein–protein interaction network analysis for plasma proteins associated with polygenic scores (for Figure 4i)

**Statistic**	**Value**
Number of nodes	19<br>(14 + 5 interactors)
Number of edges	113
Average node degree	11.9
Average local clustering coefficient	0.844
Expected number of edges	22
Protein–protein interaction enrichment *p*-value	<1.0E−16

Supplementary Table 24. Cell-type enrichment analysis of the plasma proteins in each cluster (for Figure 5c)

	Cluster 1			Cluster 2		
	Tissue-specific genes	Fold change	*p*-value	Tissue-specific genes	Fold change	*p*-value
**B cells**	10	2.8407	**0.0195**	6	0.8338	0.8041
Dendritic cells	7	1.0749	0.9769	11	0.8263	0.8041
Endothelial cells	7	0.5681	0.9769	27	1.0720	0.5852
Eosinophils	4	0.5588	0.9769	23	1.5719	0.0489
**Erythroblasts**	2	0.5980	0.9769	18	2.6331	**0.0003**
Macrophages	5	0.6360	0.9769	20	1.2445	0.3372
**Megakaryocytes**	3	0.5063	0.9769	28	2.3116	**0.0001**
Monocytes	5	0.7222	0.9769	12	0.8479	0.8041
Neutrophils	7	1.2174	0.9769	15	1.2762	0.3372
Natural killer cells	4	1.1555	0.9769	10	1.4132	0.3372
T cells	3	1.1363	0.9769	5	0.9265	0.8041
	**Cluster 3**			**Cluster 4**		
	Tissue-specific genes	Fold change	*p*-value	Tissue-specific genes	Fold change	*p*-value
**B cells**	1	0.5366	0.9426	13	2.7295	**0.0033**
**Dendritic cells**	4	1.1602	0.9426	19	2.1564	**0.0033**
Endothelial cells	7	1.0731	0.9426	13	0.7799	0.8826
**Eosinophils**	3	0.7917	0.9426	18	1.8587	**0.0183**
Erythroblasts	1	0.5648	0.9426	4	0.8841	0.7466
Macrophages	3	0.7208	0.9426	14	1.3162	0.3013
Megakaryocytes	2	0.6375	0.9426	9	1.1226	0.5629
Monocytes	4	1.0913	0.9426	11	1.1744	0.5183
Neutrophils	4	1.3141	0.9426	8	1.0284	0.6417
Natural killer cells	2	1.0913	0.9426	8	1.7082	0.1940
T cells	0	0.0000	1.0000	7	1.9597	0.1625

Cell types with significant enrichment of the identified plasma proteins ($p < 0.05$) are displayed in bold text.

Supplementary Table 25. Plasma proteins classified in distinct clusters are enriched in specific blood cell types (for Figures 5c, d)

	Cluster 1	Cluster 2		Cluster 4		
**Index**	**B cells**	**Erythroblasts**	**Megakaryocytes**	**B cells**	**Dendritic cells**	**Eosinophils**
1	CD27	CRADD	PARK7	CD69	IRF9	AXIN1
2	IFNLR1	PARK7	ARHGAP1	LY9	GLB1	IRF9
3	FAM3C	CCT5	QDPR	SIT1	VIM	BACH1
4	TNF	GLO1	CCT5	ADA	NFKBIE	CBL
5	CD38	ARHGEF12	GLO1	BACH1	ZBTB17	ICAM3
6	IGLC2	EIF4G1	USO1	LAT2	PLAU	TREML2
7	IL4R	SOD1	ARHGEF12	HSP90B1	VCAM1	SIRPA
8	SERPINA9	PRKAB1	EIF4G1	TOP2B	CDKN1A	TNFSF14
9	TCL1A	AKT1S1	SOD1	NFKBIE	COCH	ZBTB17
10	TNFRSF10A	ATG4A	CASP2	ZBTB17	DAPP1	NFATC1
11		BLVRB	MAX	NFATC1	ICAM1	ADAM8
12		FOXO3	TXLNA	CD22	IL15	CLEC4C
13		HAGH	CLIP2	FOXO1	IL15RA	LRPAP1
14		HMBS	MANF		IL6	SEMA7A
15		METAP2	YES1		LAP3	TGFA
16		PSMD9	GOPC		NUB1	WWP2
17		UBAC1	EIF4EBP1		TANK	ARSB
18		EIF4EBP1	ENO2		ARSB	HS6ST1
19			GP6		FOXO1	
20			HEXIM1			
21			ITGA6			
22			LAT			
23			NT5C3A			
24			PLXNA4			
25			PMVK			
26			PRTFDC1			
27			SH2B3			
28			SRC			

Supplementary Table 26. Summary of protein–protein interaction network analysis for plasma proteins enriched in specific blood cell types (for Figures 5d)

**Statistic/cell type**	**B cells**	**Dendritic cells**	**Eosinophils**	**Erythroblasts**	**Megakaryocytes**
Number of nodes	22	19	18	18	28
Number of edges	64	50	20	41	58
Average node degree	5.82	5.26	2.22	4.56	4.14
Average local clustering coefficient	0.646	0.665	0.369	0.678	0.558
Expected number of edges	23	21	6	19	40
Protein–protein interaction enrichment *p*-value	1E−12	5.34E−08	5.1E−06	7.82E−06	0.00501

Supplementary Table 27. Transcript levels of plasma proteins that are abundant in B cells (for Figures 5d, e)

Gene symbol	B cells	Dendritic cells	Endothelial cells	Eosinophils	Erythroblasts	Macrophages
SERPINA9	30.0	0.0	0.0	0.0	0.0	0.0
IGLC2	9998.7	1.3	0.0	11.1	0.1	0.0
TCL1A	246.1	0.3	0.0	1.4	0.0	0.0
CD22	422.0	2.1	0.1	4.3	0.1	13.2
CD38	64.5	5.5	0.2	0.5	0.0	4.4
CD27	52.5	0.0	0.0	1.8	0.0	0.0
SIT1	22.6	0.4	0.0	1.7	0.1	2.0
IFNLR1	9.4	1.9	0.0	0.1	0.0	11.6
LY9	47.8	1.9	0.0	2.6	0.3	10.9
IL4R	163.4	84.4	28.9	94.0	8.3	21.4
FAM3C	46.5	15.9	31.8	0.9	7.2	13.6
CD69	297.3	8.5	1.6	45.4	35.4	0.0
ADA	29.8	2.4	13.8	0.6	5.8	11.5
TNF	12.8	11.8	0.0	0.7	0.4	1.7
LAT2	124.1	18.7	0.4	20.2	10.9	39.0
NFATC1	18.0	3.5	8.9	14.8	4.5	3.6
NFKBIE	37.7	26.7	14.2	19.4	2.9	37.6
FOXO1	28.2	39.8	30.6	4.4	6.1	2.7
HSP90B1	415.3	186.1	500.0	56.7	125.5	273.8
ZBTB17	34.0	34.1	13.3	25.5	11.9	15.8
TNFRSF10A	10.4	4.2	9.4	8.4	3.3	11.5
BACH1	64.4	25.4	10.3	83.0	38.5	38.3
TOP2B	103.4	72.5	62.4	67.6	57.1	34.6

Gene symbol	Megakaryocytes	Monocytes	Neutrophils	Natural killer cells	T cells
SERPINA9	0.0	0.0	0.0	0.0	0.0
IGLC2	0.0	0.7	27.5	0.4	5.8
TCL1A	0.0	0.3	0.5	0.4	0.0
CD22	0.4	0.7	0.3	0.2	0.1
CD38	0.0	2.8	7.1	24.9	3.9
CD27	0.0	0.1	0.3	0.4	60.8
SIT1	0.0	0.1	0.2	0.1	29.2
IFNLR1	0.0	0.0	0.0	0.1	0.4
LY9	0.1	0.8	0.1	24.5	43.4
IL4R	15.4	26.2	87.1	10.7	45.7
FAM3C	23.3	4.4	1.4	17.7	4.8
CD69	261.3	21.7	4.9	117.3	367.5
ADA	8.6	3.6	2.3	25.1	13.9
TNF	6.7	7.1	3.7	1.1	4.7
LAT2	69.4	115.4	64.5	76.3	1.6
NFATC1	10.9	2.7	1.9	5.9	8.0
NFKBIE	5.3	22.6	4.6	4.2	4.0
FOXO1	7.9	8.2	7.0	4.2	24.9
HSP90B1	370.2	124.6	118.3	165.7	120.8
ZBTB17	12.1	8.7	17.7	13.5	15.5
TNFRSF10A	2.4	3.3	4.7	0.7	13.8
BACH1	40.2	86.2	82.0	11.0	13.9
TOP2B	143.6	38.0	69.4	81.6	71.2

Transcript abundance is indicated as the FPKM values obtained from the RNA-sequencing data. FPKM, fragments per kilobase per million mapped fragments.

Supplementary References

1. Jun, G. *et al.* Meta-analysis confirms CR1, CLU, and PICALM as Alzheimer disease risk loci and reveals interactions with APOE genotypes. *Arch. Neurol.* **67**, 1473–1484 (2010).

2. Naj, A. C. *et al.* Common variants at MS4A4/MS4A6E, CD2AP, CD33 and EPHA1 are associated with late-onset Alzheimer's disease. *Nat. Genet.* **43**, 436–443 (2011).

3. Lee, J. H., Cheng, R., Graff-Radford, N., Foroud, T. & Mayeux, R. Analyses of the national institute on aging late-onset Alzheimer's disease family study: Implication of additional loci. *Arch. Neurol.* **65**, 1518–1526 (2008).

4. Das, S. *et al.* Next-generation genotype imputation service and methods. *Nat. Genet.* **48**, 1284–1287 (2016).

5. Taliun, D. *et al.* Sequencing of 53,831 diverse genomes from the NHLBI TOPMed Program. *Nature* **590**, 290–299 (2021).

6. Loh, P. R., Palamara, P. F. & Price, A. L. Fast and accurate long-range phasing in a UK Biobank cohort. *Nat. Genet.* **48**, 811–816 (2016).

7. Corces, M. R. *et al.* Single-cell epigenomic analyses implicate candidate causal variants at inherited risk loci for Alzheimer's and Parkinson's diseases. *Nat. Genet.* **52**, 1158–1168 (2020).

8. Bellenguez, C. *et al.* Large meta-analysis of genome-wide association studies expands knowledge of the genetic etiology of Alzheimer's disease and highlights potential translational opportunities. *medRxiv* 2020.10.01.20200659 (2020) doi:10.1101/2020.10.01.20200659.

9. Schwartzentruber, J. *et al.* Genome-wide meta-analysis, fine-mapping, and integrative prioritization identify new Alzheimer's disease risk genes. *medRxiv* (2020) doi:10.1101/2020.01.22.20018424.

10. Wightman, D. P. *et al.* Largest GWAS (N=1,126,563) of Alzheimer's Disease Implicates Microglia and Immune Cells. *medRxiv* 2020.11.20.20235275 (2020) doi:10.1101/2020.11.20.20235275.

11. Jansen, I. E. *et al.* Genome-wide meta-analysis identifies new loci and functional pathways influencing Alzheimer's disease risk. *Nat. Genet.* (2019) doi:10.1038/s41588-018-0311-9.

12. Kunkle, B. W. *et al.* Genetic meta-analysis of diagnosed Alzheimer's disease identifies new risk loci and implicates Aβ, tau, immunity and lipid processing. *Nat. Genet.* **51**, 414–430 (2019).

13. Marioni, R. E. *et al.* GWAS on family history of Alzheimer's disease. *Transl. Psychiatry* **8**, 99 (2018).

14. Kunkle, B. W. *et al.* Novel Alzheimer Disease Risk Loci and Pathways in African American Individuals Using the African Genome Resources Panel: A Meta-analysis. *JAMA Neurol.* **78**, 102–113 (2021).

15. Jia, L. *et al.* Prediction of Alzheimer's disease using multi-variants from a Chinese genome-wide association study. *Brain* (2020) doi:10.1093/brain/awaa364.

16. Jun, G. R. *et al.* Transethnic genome-wide scan identifies novel Alzheimer's disease loci. *Alzheimer's Dement.* **13**, 727–738 (2017).
