# Supplementary material for: Deep learning-based polygenic risk analysis for Alzheimer’s disease prediction
Source: Commun Med (Lond). 2023 Apr 6;3:49. doi: 10.1038/s43856-023-00269-x (PMC10079691; doi:10.1038/s43856-023-00269-x)
Supplement: Supplementary file 3 — Description of Additional Supplementary Files [file 43856_2023_269_MOESM3_ESM.pdf]

Description of Additional Supplementary Files

File Name: Supplementary Data

Description: Supplementary Data 1–10
