# Supplementary material for: Deep learning-based polygenic risk analysis for Alzheimer’s disease prediction
Source: Commun Med (Lond). 2023 Apr 6;3:49. doi: 10.1038/s43856-023-00269-x (PMC10079691; doi:10.1038/s43856-023-00269-x)
Supplement: Supplementary file 5 — Reporting Summary [file 43856_2023_269_MOESM5_ESM.pdf]

nature research

Corresponding author(s): Nancy Y. Ip

Last updated by author(s): Jan 25, 2023

Reporting Summary

Nature Research wishes to improve the reproducibility of the work that we publish. This form provides structure for consistency and transparency in reporting. For further information on Nature Research policies, see our Editorial Policies and the Editorial Policy Checklist.

Statistics

For all statistical analyses, confirm that the following items are present in the figure legend, table legend, main text, or Methods section.

n/a	Confirmed	
☐	☒	The exact sample size (*n*) for each experimental group/condition, given as a discrete number and unit of measurement
☐	☒	A statement on whether measurements were taken from distinct samples or whether the same sample was measured repeatedly
☐	☒	The statistical test(s) used AND whether they are one- or two-sided<br>*Only common tests should be described solely by name; describe more complex techniques in the Methods section.*
☐	☒	A description of all covariates tested
☐	☒	A description of any assumptions or corrections, such as tests of normality and adjustment for multiple comparisons
☐	☒	A full description of the statistical parameters including central tendency (e.g. means) or other basic estimates (e.g. regression coefficient) AND variation (e.g. standard deviation) or associated estimates of uncertainty (e.g. confidence intervals)
☐	☒	For null hypothesis testing, the test statistic (e.g. *F*, *t*, *r*) with confidence intervals, effect sizes, degrees of freedom and *P* value noted<br>*Give P values as exact values whenever suitable.*
☒	☐	For Bayesian analysis, information on the choice of priors and Markov chain Monte Carlo settings
☒	☐	For hierarchical and complex designs, identification of the appropriate level for tests and full reporting of outcomes
☐	☒	Estimates of effect sizes (e.g. Cohen's *d*, Pearson's *r*), indicating how they were calculated

Our web collection on statistics for biologists contains articles on many of the points above.

Software and code

Policy information about availability of computer code

Data collection

The genotype data for AD cohorts (ADC, LOAD) were retrieved from dbGaP, ADNI dataset was retrieved from ADNI consortium. For in-house whole-genome sequencing data, 15G raw data per individual was generated using the illumina platform and reported in previous publication (please refer to the corresponding Methods section).

All data associated with this study are in the main text and the Supplementary Information or Supplementary Data. The Supplementary Data 1-10 can be found in the Supplementary Data file as separated spreadsheets. The genotype data used in the study for variant selection can be accessed in the corresponding sources: the National Institute on Aging–Late Onset Alzheimer's Disease Family Study cohort (LOAD) raw data can be accessed in the database of Genotypes and Phenotypes (dbGaP) at phs000168.v2.p2; the Alzheimer's Disease Genetics Consortium (ADGC) Genome Wide Association Study–NIA Alzheimer's Disease Centers cohort (ADC) raw data can be accessed in the dbGaP at phs000372.v1.p1; and the Alzheimer's Disease Neuroimaging Initiative cohort (ADNI) dataset can be accessed in the ADNI database (https://adni.loni.usc.edu/).

The genetic and AD-associated endophenotypic data analysis results are provided in the Supplementary Information. For data from the Chinese population, the consent form signed by individual participants states that the research content will be kept private under the supervision of the hospital and research team. Therefore, these data will be made available and shared only in the context of a formal collaboration; applications for data sharing and project collaboration will be processed and reviewed by a Review Panel hosted at the Hong Kong University of Science and Technology. Researchers may contact sklneurosci@ust.hk for further details on project collaboration and the sharing of the data from this study.

Data analysis

Sequencing data were subjected to FastQC (www.bioinformatics.babraham.ac.uk/projects/fastqc/) to check quality and Trimmomatic to trim and filter low-quality reads. Subsequent analysis was conducted by subjecting data to the GotCloud pipeline with data processing and variant detection using the default settings. The clean genotype files to Beagle and Thunder for genotype refinement. R program was used for

statistical analysis. Please refer the Methods and Supplemental Methods section for details.

The code for the neural network for polygenic score analysis (NNP), together with the dummy datasets have been all deposited at GitHub (https://github.com/xzhouai/NNP; DOI: 10.5281/zenodo.7566919).

For manuscripts utilizing custom algorithms or software that are central to the research but not yet described in published literature, software must be made available to editors and reviewers. We strongly encourage code deposition in a community repository (e.g. GitHub). See the Nature Research guidelines for submitting code & software for further information.

Data

Policy information about availability of data

All manuscripts must include a data availability statement. This statement should provide the following information, where applicable:

- Accession codes, unique identifiers, or web links for publicly available datasets
- A list of figures that have associated raw data
- A description of any restrictions on data availability

The AD GWAS data used in the study for variant selection can be accessed in the corresponding manuscripts: the National Institute on Aging—Late Onset Alzheimer's Disease Family Study (LOAD) raw data can be accessed in dbGaP at phs000168.v2.p2; the Alzheimer's Disease Genetics Consortium (ADGC) Genome Wide Association Study—NIA Alzheimer's Disease Centers Cohort (ADC) raw data can be accessed in dbGaP at phs000372.v1.p1; the Alzheimer's Disease Neuroimaging Initiative (ADNI) dataset can be accessed at ADNI database (http://adni.loni.usc.edu/).
The genetic and AD-associated endophenotype data analysis results are given in the Supplementary Information. For raw data, the consent form signed by individual participants stated that the research content will be kept private under supervision of the hospital and research team. Therefore, these data will be made available and shared only in the context of a formal collaboration; applications for data sharing and project collaboration will be processed and reviewed by a Review Panel hosted at the Hong Kong University of Science and Technology. Researchers may contact [sklneurosci@ust.hk] for further details on project collaboration and sharing of the data from this study.

Field-specific reporting

Please select the one below that is the best fit for your research. If you are not sure, read the appropriate sections before making your selection.

☒ Life sciences ☐ Behavioural & social sciences ☐ Ecological, evolutionary & environmental sciences

For a reference copy of the document with all sections, see nature.com/documents/nr-reporting-summary-flat.pdf

Life sciences study design

All studies must disclose on these points even when the disclosure is negative.

Sample size	The sample size of different cohorts were described in Methods and in Supplementary Table 1. For analysis, the sample size has listed in the corresponding legends.
Data exclusions	Individuals exerted gender missingness/inconsistency, relativeness (IBD) or deviated from the main population (PCA outliers) were excluded from the study (Please refer to Methods original publications for details).
Replication	For evaluating the accuracy of deep learning model in classifying AD, independent samples or datasets were used as the validation datasets.
Randomization	Randomization was applied when splitting the samples into training and test datasets.
Blinding	The service provider was blinded for the data category when performing the whole-genome sequencing or MRI analysis.

Reporting for specific materials, systems and methods

We require information from authors about some types of materials, experimental systems and methods used in many studies. Here, indicate whether each material, system or method listed is relevant to your study. If you are not sure if a list item applies to your research, read the appropriate section before selecting a response.

Materials & experimental systems

n/a	Involved in the study	
☒	☐	Antibodies
☒	☐	Eukaryotic cell lines
☒	☐	Palaeontology and archaeology
☒	☐	Animals and other organisms
☐	☒	Human research participants
☒	☐	Clinical data
☒	☐	Dual use research of concern

Methods

n/a	Involved in the study	
☒	☐	ChIP-seq
☒	☐	Flow cytometry
☐	☒	MRI-based neuroimaging

Human research participants

Policy information about studies involving human research participants

Population characteristics	The data for Chinese WGS cohort 1 (N = 2,340 comprising 1,116 patients with AD, 309 patients with MCI, and age- and sex-matched 915 NCs) have been published elsewhere (Zhou et al., 2018). After quality control, data from 2,340 participants were retained for analysis. Data for Chinese WGS cohort 2 (N= 1,077 comprising 356 patients with AD, 68 patients with MCI, and 653 age- and gender-matched NCs)) have also been published elsewhere (Zhou et al., 2020).
Recruitment	Please refer to the previous publications for recruitment criteria.
Ethics oversight	The study was approved by all corresponding Research Institutes and Hospitals. All participants provided written informed consent for both study enrollment and sample collection. please refer to the previous publications for information.

Note that full information on the approval of the study protocol must also be provided in the manuscript.

Magnetic resonance imaging

Experimental design

Design type	Cross-sectional morphometry and fluid-attenuated inversion recovery analysis
Design specifications	T1-weighted structural MRI and Fluid-attenuated inversion recovery MRI were assayed to examine association between brain states and polygenic risk
Behavioral performance measures	None

Acquisition

Imaging type(s)	T1 and T2 MRI
Field strength	3T
Sequence & imaging parameters	3D FFE (gradient echo) pulse sequence was used. The images were acquired from coronal view, with slice thickness 5mm, TE/TR/flip angle = 3ms/7ms/8. The acquired image matrix size was 240 × 25 × 240.<br><br>For FLARE, the images were acquired from coronal view, with slice thickness 5mm or 1.1mm, TE/TR/flip angle = 330ms/8000ms/90 or 125ms/11000ms/90. The acquired image matrix size was 704x704x25 and 235x235x24.
Area of acquisition	Whole brain

Diffusion MRI ☐ Used ☒ Not used

Preprocessing

Preprocessing software	The MRI data were preprocessed by AccuBrain® IV1.2 (BrainNow Medical Technology Ltd), a brain quantification tool that performs brain structure and tissue segmentation and quantification in a fully automatic mode. Given the T1-weighted MRI data, several brain structures (e.g., hippocampus, lateral ventricle, amygdala, etc) and three major brain tissues (i.e., white matter, gray matter and CSF) are segmented automatically based on prior anatomical knowledge specified by experienced radiologists. The anatomical information is automatically transformed into the individual brain.
Normalization	For details please refer to Abrigo et al., Acta Radiologica 2018
Normalization template	For details please refer to Abrigo et al., Acta Radiologica 2018
Noise and artifact removal	For details please refer to Abrigo et al., Acta Radiologica 2018
Volume censoring	Not applicable

Statistical modeling & inference

Model type and settings	ROI-based approach was applied for the analysis. Additional details on statistical methods, including all model parameters can be found in Methods and Supplements Methods sections.
Effect(s) tested	We tested for associations between imaging measures and calculated polygenic score

Specify type of analysis: ☐ Whole brain ☒ ROI-based ☐ Both

Anatomical location(s): Automated labelling by Accubrain

Statistic type for inference<br>(See Eklund et al. 2016)	Neither voxel-wise or cluster-wise was used
Correction	Not applicable due to small sample size

Models & analysis

n/a	Involved in the study
☐	☒ Functional and/or effective connectivity
☐	☒ Graph analysis
☐	☒ Multivariate modeling or predictive analysis

Functional and/or effective connectivity	For protein-protein network analysis, we used the Search Tool for the Retrieval of Interacting Genes/Proteins (STRING) database to investigate the Functional connectivity of identified plasma proteins.
Graph analysis	For modeling the disease risk, we applied the graph neural network model to the genetic data. Each participant was represented in a graph with nodes denoting the selected 37 variants, edges denoting the pairwise LD (calculated by PLINK) among the variants, and graph labels denoting the phenotypes. For node features, in addition to the allele dosage, we considered the biological properties of variants, including whether they resided in coding or untranslated regions, and the number of events of histone, open chromatin, polymerase, and transcription factor binding.
Multivariate modeling and predictive analysis	We used the artificial neural network and graph neural network models, which took the genetic information from multiple variants as input to classify the individual disease phenotype (alzheimer's disease)
